# Harnessing antifungal immunity in pursuit of a *Staphylococcus aureus* vaccine strategy

**DOI:** 10.1371/journal.ppat.1008733

**Published:** 2020-08-20

**Authors:** Marissa J. Paterson, JR Caldera, Christopher Nguyen, Purnima Sharma, Anthony M. Castro, Stacey L. Kolar, Chih-Ming Tsai, Jose J. Limon, Courtney A. Becker, Gislâine A. Martins, George Y. Liu, David M. Underhill

**Affiliations:** 1 F. Widjaja Foundation Inflammatory Bowel & Immunobiology Research Institute, and the Division of Immunology, Department of Biomedical Sciences, Cedars-Sinai Medical Center, Los Angeles, California, United States of America; 2 Division of Pediatric Infectious Diseases and Research Division of Immunology, Department of Biomedical Sciences, Cedars-Sinai Medical Center, Los Angeles, California, United States of America; 3 Division of Infectious Diseases, Department of Pediatics, UCSD, San Diego, California, United States of America; 4 Department of Medicine, Division of Gastroenterology, Cedars-Sinai Medical Center, Los Angeles, California, United States of America; Tulane University School of Medicine, UNITED STATES

## Abstract

*Staphylococcus aureus* (*S*. *aureus*) is one of the most common bacterial infections worldwide, and antibiotic resistant strains such as Methicillin-Resistant *S*. *aureus* (MRSA) are a major threat and burden to public health. MRSA not only infects immunocompromised patients but also healthy individuals and has rapidly spread from the healthcare setting to the outside community. However, all vaccines tested in clinical trials to date have failed. Immunocompromised individuals such as patients with HIV or decreased levels of CD4^+^ T cells are highly susceptible to *S*. *aureus* infections, and they are also at increased risk of developing fungal infections. We therefore wondered whether stimulation of antifungal immunity might promote the type of immune responses needed for effective host defense against *S*. *aureus*. Here we show that vaccination of mice with a fungal β-glucan particle (GP) loaded with *S*. *aureus* antigens provides protective immunity to *S*. *aureus*. We generated glucan particles loaded with the four *S*. *aureus* proteins ClfA, IsdA, MntC, and SdrE, creating the 4X-SA-GP vaccine. Vaccination of mice with three doses of 4X-SA-GP promoted protection in a systemic model of *S*. *aureus* infection with a significant reduction in the bacterial burden in the spleen and kidneys. 4X-SA-GP vaccination induced antigen-specific Th1 and Th17 CD4^+^ T cell and antibody responses and provided long-term protection. This work suggests that the GP vaccine system has potential as a novel approach to developing vaccines for *S*. *aureus*.

## Author summary

*Staphylococcus aureus* (*S*. *aureus*) is a serious burden to public health as it is a leading cause of antibiotic-resistant infections worldwide. Vaccines aimed at targeting *S*. *aureus* have failed in clinical trials, and the reason for this lack of success remains unclear. As this pathogen continues to rapidly spread on a global scale, it is vital that new approaches to *S*. *aureus* vaccination are developed. In this study, we show that activation of antifungal immunity with a β-glucan particle vaccine can be harnessed to direct protection against *S*. *aureus* systemic infections in mice. This work broadens the use of the β-glucan particle vaccine system for potential use as a much-needed vaccine targeting *S*. *aureus*.

## Introduction

*Staphylococcus aureus* is both a human commensal and a formidable opportunistic pathogen. As a commensal, *S*. *aureus* can be found in places such as the anterior nares, throat, skin, and gastrointestinal tract [1–3]. It is estimated that between 20–40% of individuals in the general population are colonized with *S*. *aureus* in the nasal mucosa [2, 4]. As an opportunistic pathogen, *S*. *aureus* can cause a range of diseases including skin and soft tissue infections (SSTIs), endocarditis, sepsis, pneumonia, osteomyelitis, bacteremia, and abscesses in organ tissues [5]. Moreover, *S*. *aureus* bacteremia mortality remains at a high rate of 15–50%, despite the use of novel antibiotics and emphasis on disease surveillance and prevention [6]. Antibiotic-resistant strains of *S*. *aureus* are becoming increasingly prevalent, with the most common, methicillin-resistant *S*. *aureus* (MRSA), emerging in hospitals and communities around the world. These strains infect healthy individuals and immunocompromised patients alike [1].

Unlike many bacterial infections, *S*. *aureus* does not usually promote robust protective immune responses, and individuals are not typically protected from subsequent *S*. *aureus* infections [7]. Hence, the development of a vaccine to target *S*. *aureus* is an urgent and crucial public health need. Unfortunately, vaccines tested in clinical trials thus far have proved unsuccessful in providing protection [8–11]. These vaccines focused primarily on generating antibody responses, and their failures, together with the observation that patients with defects in humoral immunity do not generally exhibit increased susceptibility to *S*. *aureus* infections [12, 13], suggest that cell-mediated immunity (CMI) may be more important than previously anticipated for vaccine-induced protection [7, 14].

Individuals that have defects in CMI such as HIV, defective IFNγ production, or treatment with high levels of glucocorticoids experience more *S*. *aureus* infections [7]. Patients with STAT3 mutations, which causes hyper-IgE (Job’s) syndrome, have dysfunctional or absent T-helper (Th)17 cells, and they are highly susceptible to *S*. *aureus* infections [15, 16]. Due to the involvement of Th17 cells in activating and recruiting neutrophils, it is not surprising that patients with neutrophil disorders are at more risk of becoming infected with *S*. *aureus* [7]. Intriguingly, many of the immune defects that predispose individuals to *S*. *aureus* also predispose individuals to fungal infections. Patients with HIV are more susceptible to fungal infections, especially chronic mucocutaneous candidiasis (CMC) [17]. Furthermore, patients with STAT3 deficiency and hyper-IgE syndrome often experience CMC along with severe *S*. *aureus* infections.

Th1 and Th17 subsets have been shown to be especially vital for antifungal responses in animal models and in humans [18–20]. Th1 and Th17 T cell subsets participate in promoting activation of phagocytes and recruitment of neutrophils via secretion of the cytokines IFNγ and IL-17 and they are also critical in clearing *S*. *aureus* infections [21]. Failure of these responses to elicit long term immunity, however, suggests that activation of other innate responses may be necessary to promote Th1 and Th17 responses during *S*. *aureus* infection. Antifungal immunity might be harnessed to activate such responses.

Innate pattern recognition receptors (PRRs), which recognize pathogen associated molecular patterns (PAMPs), are responsible for initial sensing of pathogens and tailor inflammatory immune responses to direct ensuing adaptive responses [22]. One such PAMP is β-glucan, which consists of carbohydrate polymers (β(1→3)- and β(1→6)-linked) abundantly found in most fungal cell walls [23]. β-glucans are recognized predominantly by the PRR Dectin-1 [24] as well as Complement Receptor 3 (CR3) via activation of the alternative pathway of complement [24, 25]. β-glucan particles (GPs) have been investigated as a potentially novel vaccine delivery platform [25–30]. β-glucan particles derived from the yeast *Saccharomyces cerevisiae* can act as an adjuvant by stimulating Dectin-1 and CR3. The hollow structure of GPs allows them to be loaded with payloads such as proteins or peptide antigens, DNA, siRNA, nanoparticles, or other small molecules for diverse purposes, including vaccine development [27]. Antigen-loaded glucan particles ultimately promote antigen presenting cell (APC) maturation and initiation of adaptive immune responses, with antibody production and polarization of CD4^+^ T cells to Th1 and Th17 subsets [26, 29, 31–37].

Given the connection between patients with specific immune defects and their increased risk for fungal and *S*. *aureus* infections, we hypothesized that a glucan particle vaccine could provide protective immunity to *S*. *aureus* by activating antifungal-tailored immunity though the β-glucan particle while delivering *S*. *aureus* antigens. Here we demonstrate that immunization of mice with a GP + *S*. *aureus* antigen vaccine protects mice against systemic infection with *S*. *aureus*. Multiple immune cell types including dendritic cells phagocytose the vaccine *in vivo* in the peritoneal cavity, and these professional antigen presenting cells are vital for stimulating strong adaptive T cell responses [38]. Moreover, this vaccination promotes polarization of antigen-specific CD4^+^ T cells to the Th1 and Th17 subsets and the production of antigen-specific antibodies, consistent with previous studies using ovalbumin-loaded GPs [26, 29]. While antibodies produced in response to the vaccine may provide some modest protection against infection, we determined that in our infection model, CD4^+^ T cells are critical for vaccine-induced protection. Finally, the GP + *S*. *aureus* antigen vaccine elicited long-term protection as mice had reduced bacterial burden in the spleen and kidneys and detectable antibody levels eight weeks after immunization.

## Results

The ideal number of antigens that should be included in a *S*. *aureus* vaccine is still a matter of debate [7, 14, 39, 40]. Many investigators favor a multi-antigen vaccine with the idea that this approach can allow targeting of multiple *S*. *aureus* virulence factors and survival mechanisms that may be effective at stopping the bacteria at different stages and sites of infection [7]. We have thus opted to evaluate a multi-antigen GP vaccine. The Schneewind laboratory previously tested antigenicity of surface proteins conserved within eight different *S*. *aureus* strains and identified antigens promoting the best protection [41]. Among these antigens were the surface proteins clumping factor A (ClfA), iron-regulated surface determinant protein A (IsdA), and serine-aspartate repeat-containing protein E (SdrE). Each of these antigens has also been used by others in various mouse *S*. *aureus* vaccination studies providing protection from lethal i.v. challenge, abscess formation, arthritis, and necrotic wound infection ([41–45]). Additionally, many other groups have shown the importance of another surface protein, manganese transport protein C (MntC), in *S*. *aureus* virulence and the efficacy of incorporating it in vaccines for inducing protection [9, 46, 47]. As discussed further below, a couple of these antigens have progressed to study in human clinical trials. Together the data suggest that these are widely acceptable antigen targets.

We recombinantly produced and purified ClfA, IsdA, MntC, and SdrE and loaded them into our GPs to create “4X-SA-GP” for evaluation as a novel *S*. *aureus* vaccine in mice (S1 Fig). For some experiments, the four recombinant proteins were labeled with the fluorescent probe fluorescein isothiocyanate (FITC) and then incorporated into the GPs to create “FITC-4X-SA-GP”.

### 4X-SA-GP behave similarly to OVA-loaded GPs and are efficiently phagocytosed by dendritic cells and promote dendritic cell maturation and production of pro-inflammatory cytokines *in vitro*

In order to test antigen-loaded glucan particles (GPs) in the context of a *S*. *aureus* vaccine, we first validated the GP vaccine strategy using GPs loaded with ovalbumin (GP-OVA) as previously created and described by the Levitz and Ostroff groups [25, 26, 29]. GPs have immunostimulatory properties as an adjuvant, and they can also act as an antigen delivery system [27, 48–50]. We utilized a co-culture assay to confirm that the antigen we encapsulated within the GPs can be processed and presented by APCs to CD4^+^ T cells (S2 Fig). Mouse bone marrow-derived dendritic cells (BMDCs) were fed GPs loaded with varying concentrations of OVA and the BMDCs were then co-cultured with naïve OT-II CD4^+^ T cells, which recognize OVA peptide in the context of MHC-II. If the OVA antigen is eaten, processed, and presented to the OTII T cells as OVA peptide by the BMDCs, the T cells should be activated by their cognate antigen, proliferate, and polarize to specific T cell subsets based on the cytokine milieu. Assessment of the OT-II T cell cytokine production via flow cytometry revealed that the cells produced IL-17 and IFNγ, with an increase in IL-17 production corresponding with increasing concentrations of OVA in the GPs (S2A, S2C and S2D Fig). These results were also observed in the co-culture supernatants (S2F and S2G Fig). Moreover, GPs without antigen inside (GP-No OVA) did not elicit OT-II T cell proliferation, while the GPs encapsulated with OVA (0.5μg, 5 μg, 50 μg) promoted robust OT-II proliferation as determined by CFSE dilution (S2B and S2E Fig). These results establish that we were able to successfully generate antigen-loaded GPs similar to those that have been previously described [29, 51] and that these particles worked as an antigen delivery system to drive proliferation and cytokine production by OT-II CD4^+^ T cells. Moreover, the GP-OVA system specifically polarized naïve OT-II T cells into Th1 and Th17 subsets, which are the subsets most necessary to generate protection against *S*. *aureus* infection [21].

**Fig 1:**
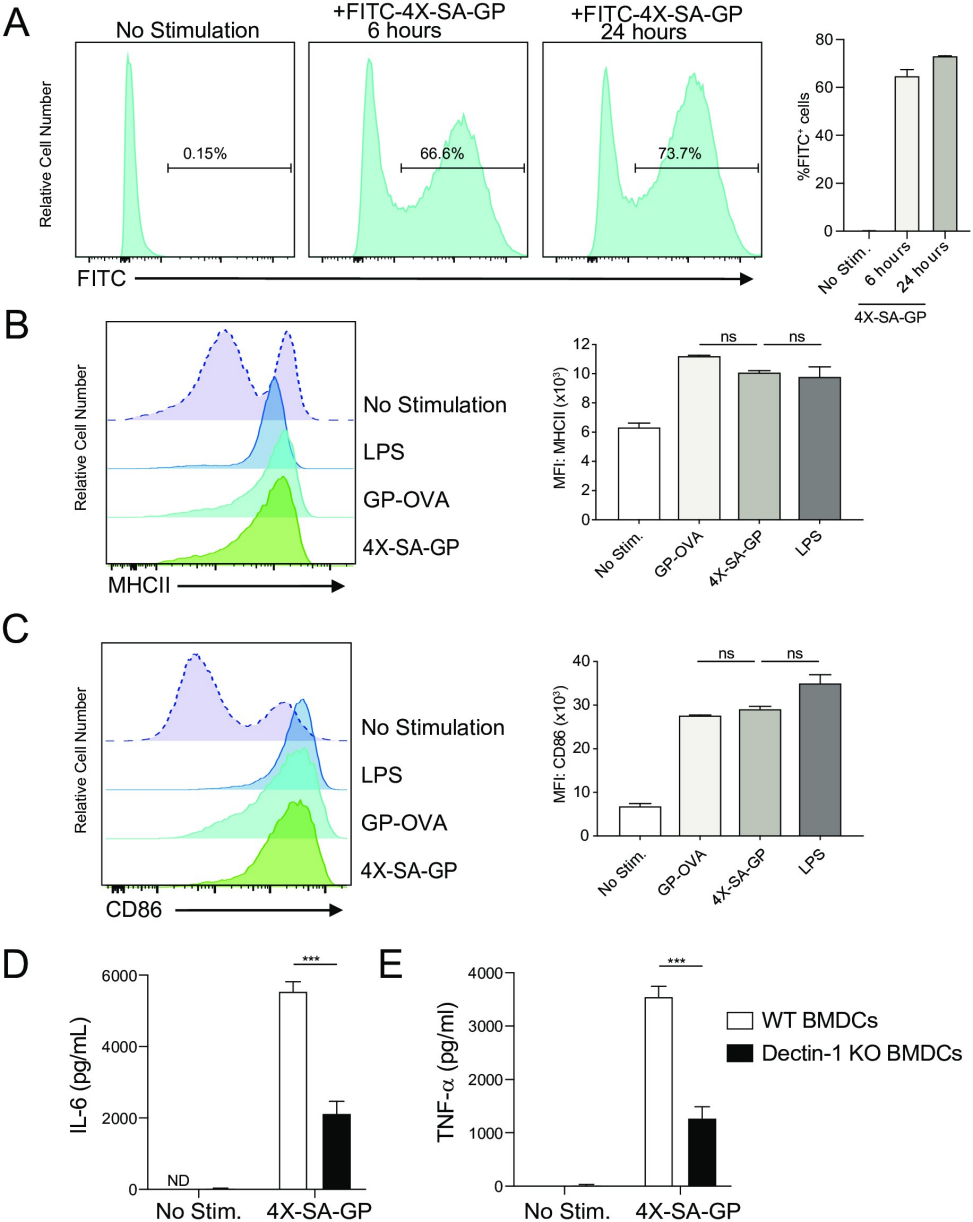
4X-SA-GP are efficiently phagocytosed by dendritic cells and promote dendritic cell maturation and production of pro-inflammatory cytokines *in vitro*. (A). Representative flow cytometry histograms showing the percentage of FITC^+^ bone marrow-derived dendritic cells (BMDCs) after being stimulated with FITC-labeled 4X-SA-GP for 6 and 24 hours. Percentages and standard deviation are representative of 2 replicates/stimulus. Flow cytometry analysis of surface MHC-II (B) and CD86 (C) on BMDCs stimulated for 24 hours with LPS, GP-OVA, and 4X-SA-GP. Data are quantified as the mean fluorescence intensity (MFI) for each marker. (D) Wild-type and (E) Dectin-1 knockout (Dectin-1 KO) BMDCs were stimulated with 4X-SA-GP and supernatants were harvested at 24 hours for cytokine determination by ELISA. Percentages and standard deviation are representative of 2 replicates/stimulus (A-C) and 3 replicates/stimulus (D-E). Data analysis was performed using ANOVA for (B) and (C) and Student’s t test for (D) and (E). ***p<0.0005; ns, not significant. Data are representative of two experiments.

To establish that the GPs are efficiently phagocytosed, we fed BMDCs FITC-4X-SA-GP for 6 and 24 hours and analyzed them by flow cytometry (Fig 1A). The BMDCs efficiently phagocytosed the particles, with over 66% of the BMDCs being FITC^+^ at 6 hours and over 70% at 24 hours post-stimulation. 4X-SA-GP also induced maturation of BMDCs as seen by upregulation of surface MHC-II and CD86 (Fig 1B and 1C). The expression levels of these maturation markers were comparable to the levels seen in BMDCs stimulated with GP-OVA and LPS. Additionally, stimulation of wild-type BMDCs with 4X-SA-GP resulted in production of IL-6 (Fig 1D) and TNF-α (Fig 1E), while stimulation of Dectin-1 knockout BMDCs led to significant reduction in the production of these cytokines (Fig 1D and 1E). In the absence of serum and therefore complement in this *in vitro* system, Dectin-1 is the primary receptor for these GPs [25]. It is possible that the antigens (or co-purifying bacterial products) in the GPs may be sensed by other PPRs, resulting in detectable production of cytokines by the Dectin-1 knockout cells.

### Characterization of 4X-SA-GP distribution *in vivo* reveals uptake by multiple immune cell types

**Fig 2:**
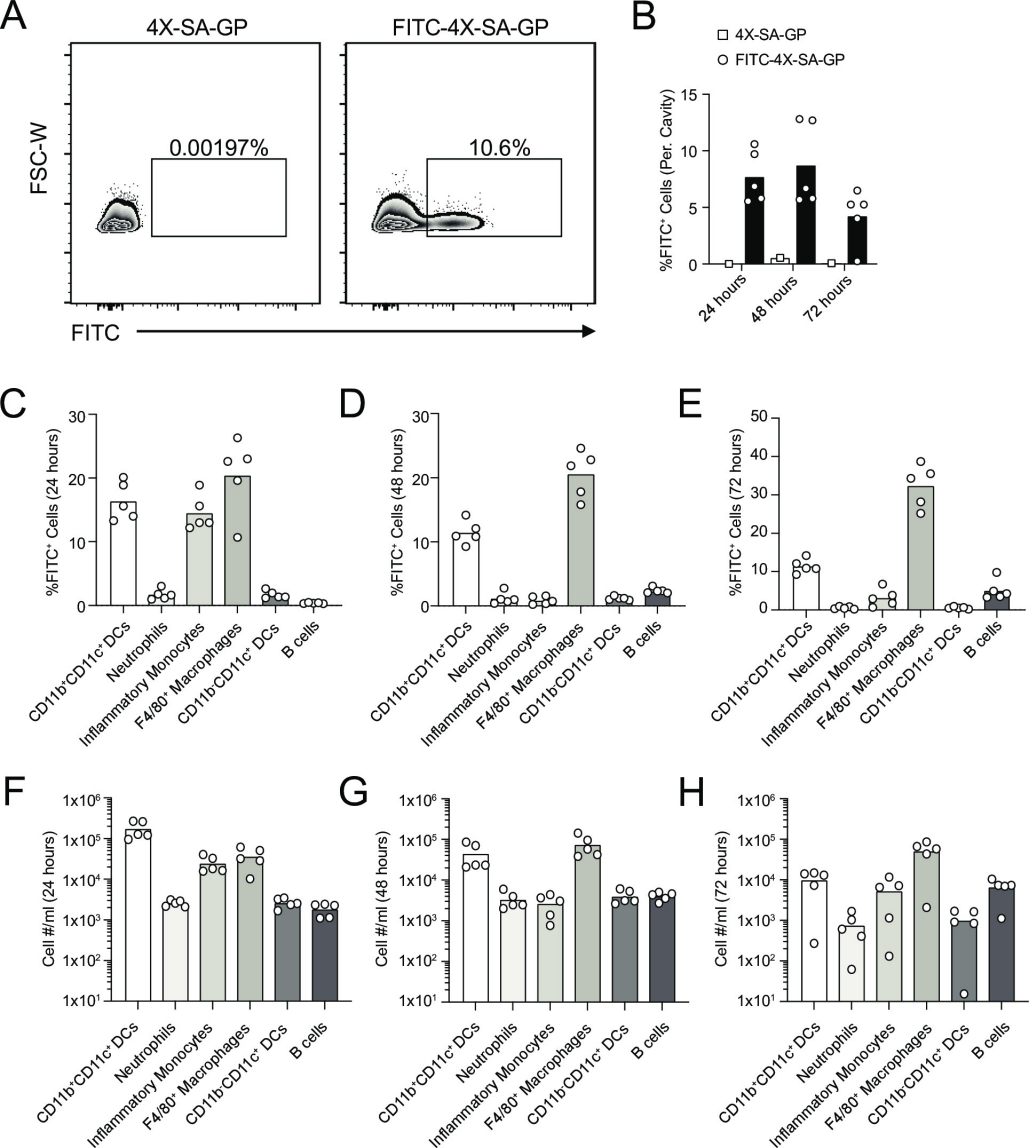
Multiple immune cell subsets phagocytose 4X-SA-GP *in vivo*. (A) Representative flow cytometry plots and gating strategy of FITC^+^ cells from the peritoneal cavity of mice injected intraperitoneally (i.p.) with 4X-SA-GP (left plot) and FITC-labeled 4X-SA-GP (right plot) at 24 hours post-injection. (B) Percentage of FITC^+^ cells in the peritoneal cavity of mice that received an i.p. injection of 4X-SA-GP or FITC-labeled 4X-SA-GP at 24, 48, and 72 hours post-injection. Each data point represents an individual mouse (n = 1 for 4X-SA-GP per time point and n = 5 for FITC-4X-SA-GP per time point). (C-E) Flow cytometry bar graphs show the frequency of the indicated immune cell subsets that are FITC^+^ cells, indicative of phagocytosing the FITC-labeled 4X-SA-GP at 24 hours (C), 48 hours (D), and 72 hours (E) post i.p. injection of the particles. (F-H) Flow cytometry bar graphs show the numbers of immune cell subsets from (C-E) that are FITC^+^ at the indicated time points, 24 hours (F), 48 hours (G), and 72 hours (H) post-injection isolated from the peritoneal cavity. Each data point represents percentages or total numbers from an individual mouse. Percentages data are representative of two experiments and total numbers data are from a single experiment.

To determine which immune cell subsets phagocytose 4X-SA-GP *in vivo*, we injected mice once with an intraperitoneal (i.p.) dose of 5x10^7^ FITC-4X-SA-GP or unlabeled 4X-SA-GP as a control. We collected cells by peritoneal lavages at 24, 48, and 72-hours post-injection and analyzed FITC^+^ immune cells by flow cytometry (Fig 2). The percent of FITC^+^ cells among the total peritoneal cavity ranged from an average of over 7.5% at 24 hours, to almost 9% at 48 hours and then dropped to a little over 4% at 72 hours post-injection (Fig 2A and 2B). Dissection of the immune subsets that were FITC^+^ revealed that myeloid DCs, neutrophils, inflammatory monocytes, F4/80^+^ macrophages, lymphoid DCs and B cells all ate the particles that were injected into the peritoneal cavity (Fig 2C–2H).

Initially, at 24 hours post-injection, myeloid DCs, inflammatory monocytes, and F4/80^+^ macrophages were the main cell types phagocytosing the 4X-SA-GPs based on FITC positivity (Fig 2C and 2F). After 48 hours, F4/80^+^ macrophages were the major cells that were FITC^+^, followed by the myeloid DCs; the percentage of FITC^+^ inflammatory monocytes had decreased substantially (Fig 2D and 2G). Interestingly, at this timepoint, the total number and percentage of FITC^+^ B cells increased (Fig 2D and 2G). Murine peritoneal cavity B cells (B-1 B cells) have been reported to have anti-microbial capabilities and are able phagocytose particulate antigen and present antigen to CD4^+^ T cells [52]. This type of murine B cell expresses CR3 [53] suggesting a plausible mechanism by which to imagine these B cells recognize and uptake the 4X-SA-GP vaccine. After 72 hours, F4/80^+^ macrophages remained the dominant FITC^+^ cell type, followed by myeloid DCs and B cells (Fig 2E and 2H).

### 4X-SA-GP vaccination induces protection from *S*. *aureus*

**Fig 3:**
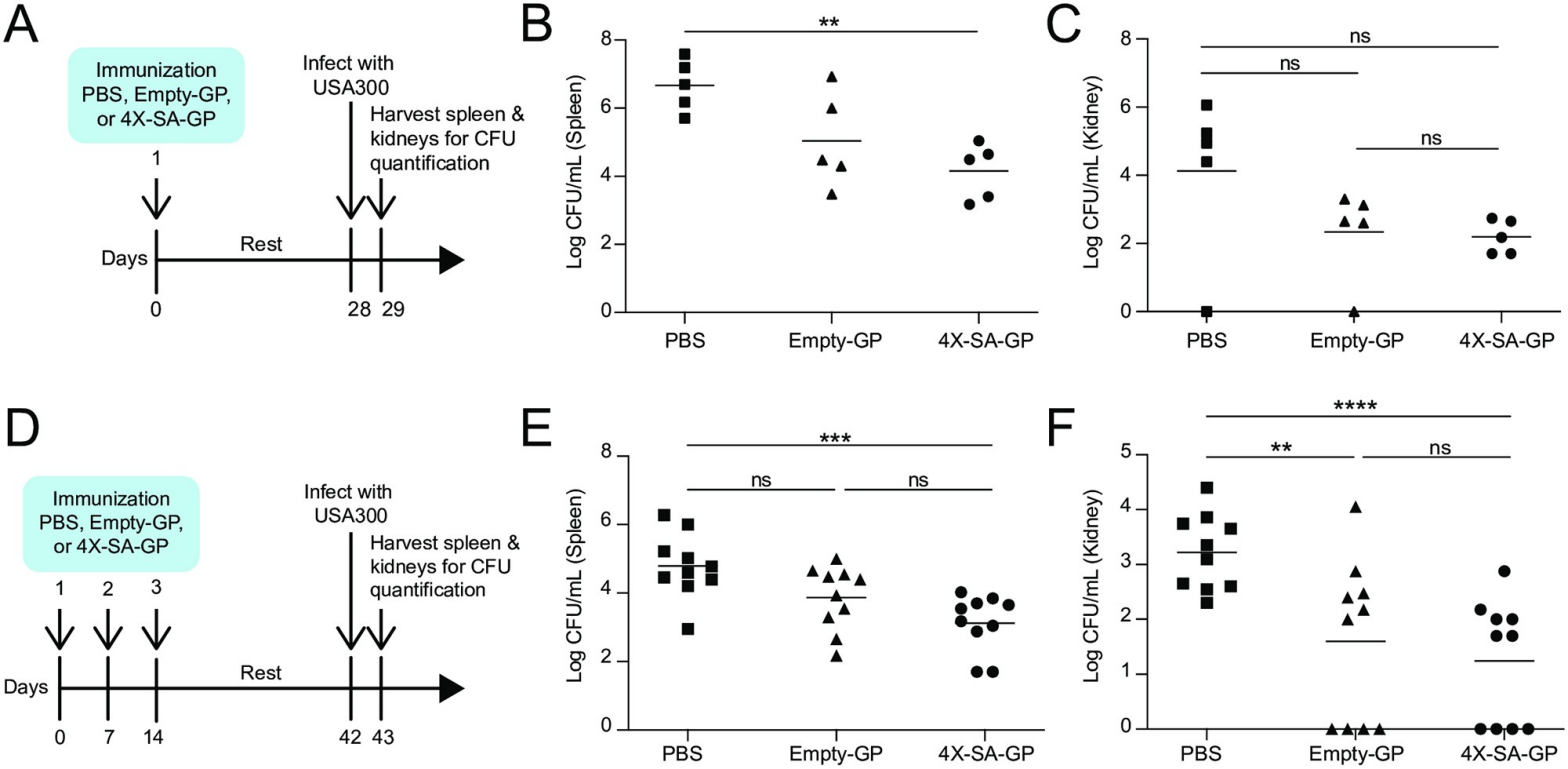
Mice immunized with 4X-SA-GP have enhanced protection against *S*. *aureus* infection. (A) Experimental timeline of 1 vaccination and *S*. *aureus* infection model. (B and C) Wild-type female mice (n = 5 per immunization group: PBS, Empty-GP, 4X-SA-GP) were infected i.p. with 2x10^7^ CFUs of *S*. *aureus* (LAC USA300) 4 weeks after the one vaccination. Bacteria from the spleen (B) and kidneys (C) were recovered after 24 hours and CFUs were enumerated. (D) Experimental timeline of 3 vaccinations and *S*. *aureus* infection model. (E and F) Wild-type female mice (n = 10 per immunization group: PBS, Empty-GP, 4X-SA-GP) were infected i.p. with 2x10^7^ CFUs of *S*. *aureus* (LAC USA300) 4 weeks after the third and final vaccination. Bacteria from the spleen (E) and kidneys (F) were recovered after 24 hours and CFUs were enumerated. Each data point represents an individual mouse. Data analysis was performed using Kruskal-Wallis test ((B) = 0.02, (C) = 0.14, (E) = 0.003, (F) = 0.001) and Mann-Whitney U test. **p<0.01, ***p<0.001, ****p<0.0005; ns, not significant. Data in (B) and (C) are representative of two experiments, data in (E) and (F) are representative of three experiments.

We tested our *S*. *aureus* vaccine *in vivo* using a systemic infection model induced by *S*. *aureus* peritoneal challenge. We vaccinated wild-type female mice with a single dose of 5x10^7^ 4X-SA-GPs (i.p.) followed by a four-week resting period (Fig 3A). Control animals were “vaccinated” with PBS or with empty-GPs. We then challenged all three groups of vaccinated mice with *S*. *aureus* (USA300) by i.p. injection of 2x10^7^ colony forming units (CFUs), and we measured bacterial burdens in the spleen and kidney after 24 hours. Mice vaccinated with 4X-SA-GP had significantly reduced bacterial burden in the spleen compared to mice vaccinated with the vehicle control (Fig 3B). The bacterial burden was also decreased in the kidneys of 4X-SA-GP immunized mice relative to the PBS control mice (Fig 3C). Although not significant, mice that received the empty-GP vaccination appeared to have diminished spleen and kidney CFUs compared to PBS control mice (Fig 3B and 3C). This could be due to the adjuvant effect of the GPs, resulting in inflammation that is still present in the peritoneal cavity at the time of infection. Alternatively, this protection may be the result of trained immunity [54–56].

We next explored the effects of increasing the vaccination schedule to three total vaccinations (Fig 3D). This approach has been suggested to provide the best adaptive memory response in mouse studies characterizing the OVA-loaded glucan particle system [29]. We vaccinated (i.p.) wild-type female mice once a week for three weeks. Four weeks after the final vaccination, we challenged the mice (i.p.) with *S*. *aureus* and analyzed bacterial burdens in the spleen and kidneys after 24 hours (Fig 3D). 4X-SA-GP vaccinated mice had significantly reduced bacterial burden in the spleen and kidneys when compared to mice that received a PBS control vaccination (Fig 3E and 3F). While the average CFU counts from the 4X-SA-GP vaccinated mice trended lower than those from the empty-GP vaccinated mice in the spleen and kidneys, they were not significantly different (Fig 3E and 3F). In fact, the empty-GP vaccination resulted in reduced bacterial burden in the kidneys that was significantly lower than that of the PBS control group (Fig 3F). These results once again point to the adjuvant quality of the glucan particles on their own and possibly of short-lived, non-antigen-specific, protective inflammatory immunity.

### 4X-SA-GP promotes antigen-specific Th1 and Th17 CD4^+^ T cell responses and strong antibody responses

**Fig 4:**
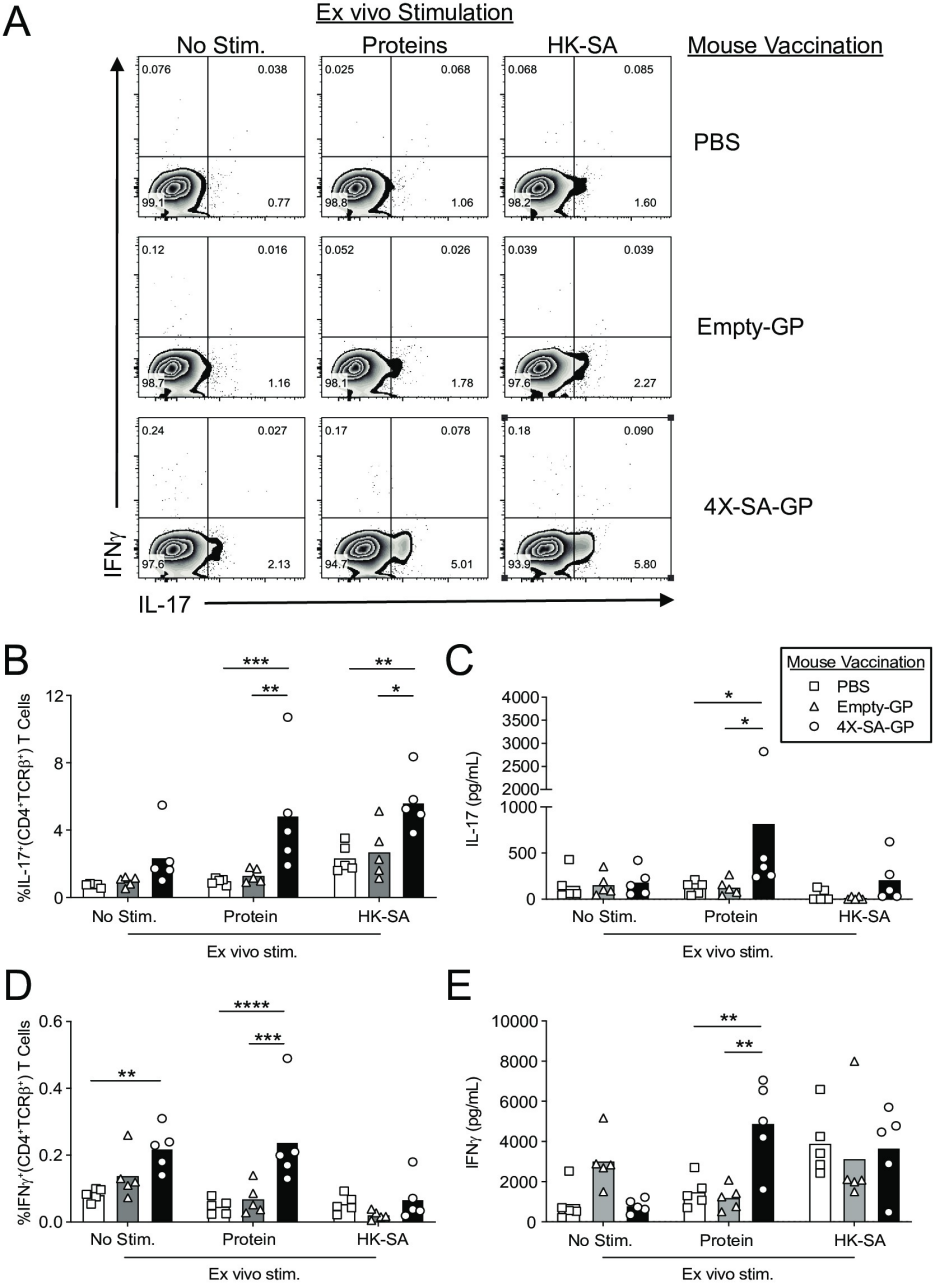
4X-SA-GP promotes antigen-specific Th1 and Th17 CD4^+^ T cell responses in mice after three immunizations. (A-E) Wild-type female mice were immunized once a week for 3 weeks with PBS (n = 5), Empty-GPs (n = 5), or 4X-SA-GP (n = 5). Five days after the final vaccination, splenocytes from each mouse were stimulated with a mixture of all 4 *S*. *aureus* purified recombinant proteins (proteins) and heat-killed *S*. *aureus* (HK-SA). After 3 days, the splenocytes were stimulated with phorbol myristate acetate (PMA) and ionomycin and then analyzed by flow cytometry after intracellular cytokine staining. Representative flow cytometry plots demonstrate the degree of IFNγ and IL-17 production from CD4^+^ T cells (gated on TCRβ^+^CD4^+^) in response to the stimuli (proteins, HK-SA) from the spleen (A). Bar graphs show the frequency of IL-17 (B) and IFNγ (D) cytokine positive cells in the spleen from the mice. Each data point represents an individual mouse in all the bar graphs. (C and E) Supernatants were harvested at day 3 from the splenocyte stimulation and the cytokines IL-17 (C) and IFNγ (E) were determined by ELISA. Data are representative of two experiments. Data analysis was performed using ANOVA. *p<0.05, **p<0.01, ***p<0.001, ****p<0.0001.

To determine if three vaccinations promoted adaptive immune responses, we assessed antigen-specific T cell responses (Fig 4). We vaccinated mice for three weeks, and five days after the final vaccination, we isolated cells from the spleens and stimulated them with a mixture of the four purified, recombinant *S*. *aureus* proteins or with heat-killed *S*. *aureus* (HK-SA). We collected supernatants after three days of culture for analysis by ELISA, and we restimulated the cells with PMA and ionomycin for analysis by intracellular cytokine staining for IFNγ and IL-17. Flow cytometry revealed a significant increase in the frequency of IL-17^+^ CD4^+^ T cells from the spleen of 4X-SA-GP vaccinated mice when stimulated with the protein mixture and with HK-SA in comparison to the CD4^+^ T cells of mice vaccinated with empty-GPs or PBS control (Fig 4A and 4B). We confirmed the increased production of IL-17 from the splenocyte culture of 4X-SA-GP vaccinated mice after stimulation with the proteins by ELISA, observing that the secreted IL-17 levels were significantly higher compared to mice immunized with empty-GPs or the PBS control (Fig 4C). Stimulation with HK-SA promoted a rise in IL-17 levels in some of the vaccinated mice, though it did not reach significance (Fig 4C).

Analysis of CD4^+^ T cells from each of the groups via flow cytometry revealed an elevated frequency of IFNγ^+^CD4^+^ T cells in the splenocyte cultures from the 4X-SA-GP vaccinated mice when stimulated with the four *S*. *aureus* recombinant proteins that was significant relative to the frequency of IFNγ^+^CD4^+^ T cells from the empty-GP vaccinated mice and PBS control mice (Fig 4A and 4D). IFNγ levels in the splenocyte supernatants were also elevated in cultures from mice vaccinated with 4X-SA-GP relative to cultures of the other two vaccination groups when stimulated with the proteins (Fig 4E). Ultimately, this assay demonstrated that immunization of mice three times with the 4X-SA-GP vaccine promoted antigen-specific Th1 and Th17 CD4^+^ T cell responses that are not seen in the empty-GP vaccination group (or the PBS control group). By comparison, single dose vaccination with 4X-SA-GP also induced antigen-specific CD4+ T cell responses, though the Th17 responses were not as strong (S3 Fig).

**Fig 5:**
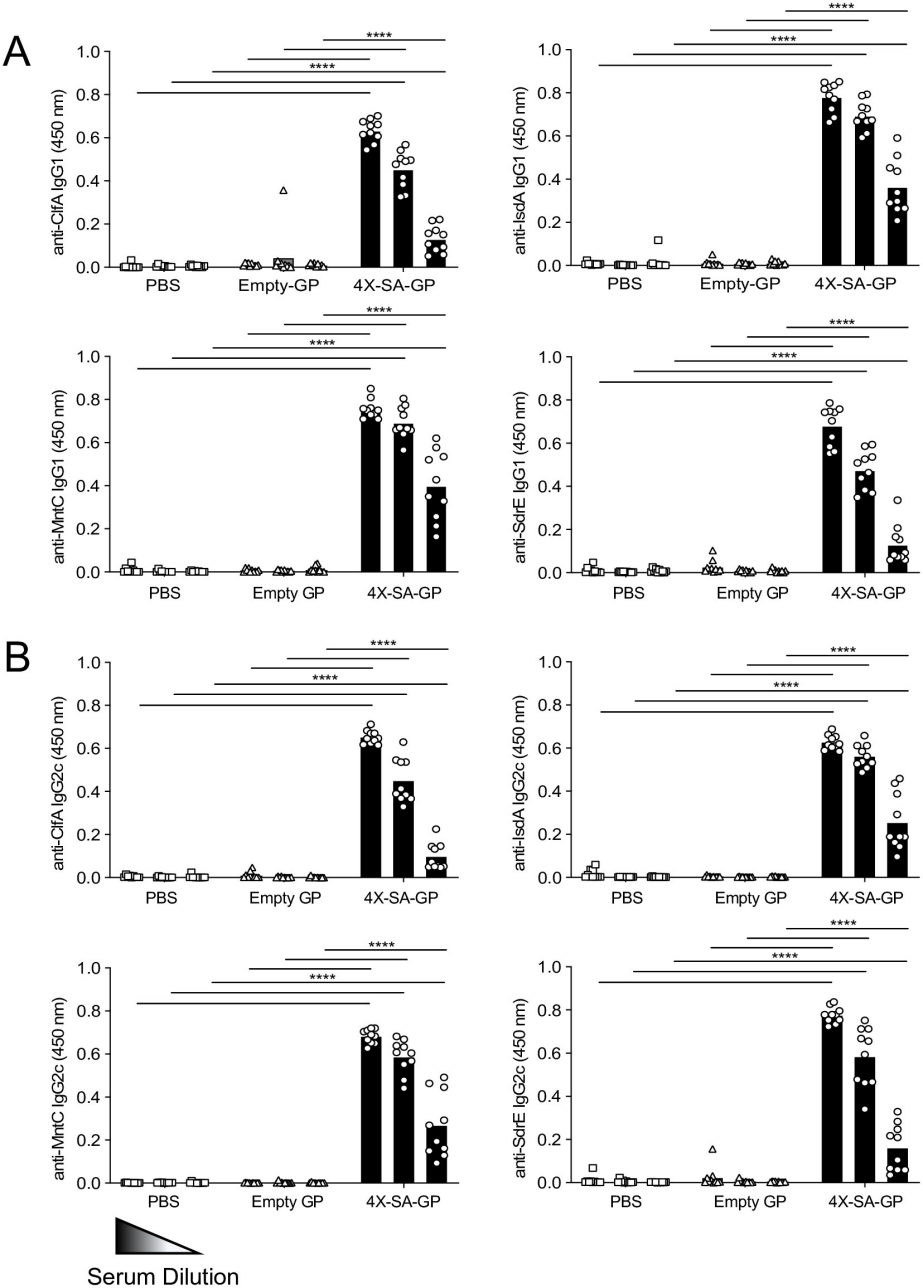
Three immunizations of mice with 4X-SA-GP induces robust antibody responses. (A and B) Serum was collected from each group of vaccinated mice 2 weeks after the third and final immunization with PBS, Empty-GP, or 4X-SA-GP (n = 10 mice per group). The serum was diluted 10X 3 times starting at 1:1000 and was then tested for antibodies specific for each of the 4 *S*. *aureus* proteins encapsulated in 4X-SA-GP by ELISA. The subclasses IgG1 (A) and IgG2c (B) were analyzed for specificity towards rClfA, rIsdA, rMntC, and rSdrE. The read-out of the assay is the optical density (OD) at 450 nm for each serum sample. Each data point represents an individual mouse. Data are representative of three experiments and were analyzed using ANOVA at each dilution. ****p<0.0001.

To further characterize the adaptive response elicited by three vaccinations with 4X-SA-GP, we investigated production of antigen-specific antibodies, specifically the IgG subclasses IgG1 and IgG2c. B cells often require help from CD4^+^ T cells to allow for isotype switching, affinity maturation, and memory [57], and therefore looking at antigen-specific antibody production can also provide some understanding into the CD4^+^ T cell response elicited by the vaccination. We detected high levels of IgG1 (Fig 5A) and IgG2c (Fig 5B) antibodies against recombinant (r)ClfA, rIsdA, rMntC, and rSdrE in the serum of all the mice vaccinated with 4X-SA-GP. All three of the serum dilutions tested for antibodies against each protein were significantly elevated compared to the serum tested from PBS or empty-GP vaccinated mice (Fig 5A and 5B). These responses are a sharp contrast to the relatively low antibody levels present in mice vaccinated with only one dose of 4X-SA-GP (S4A and S4B Fig).

**Fig 6:**
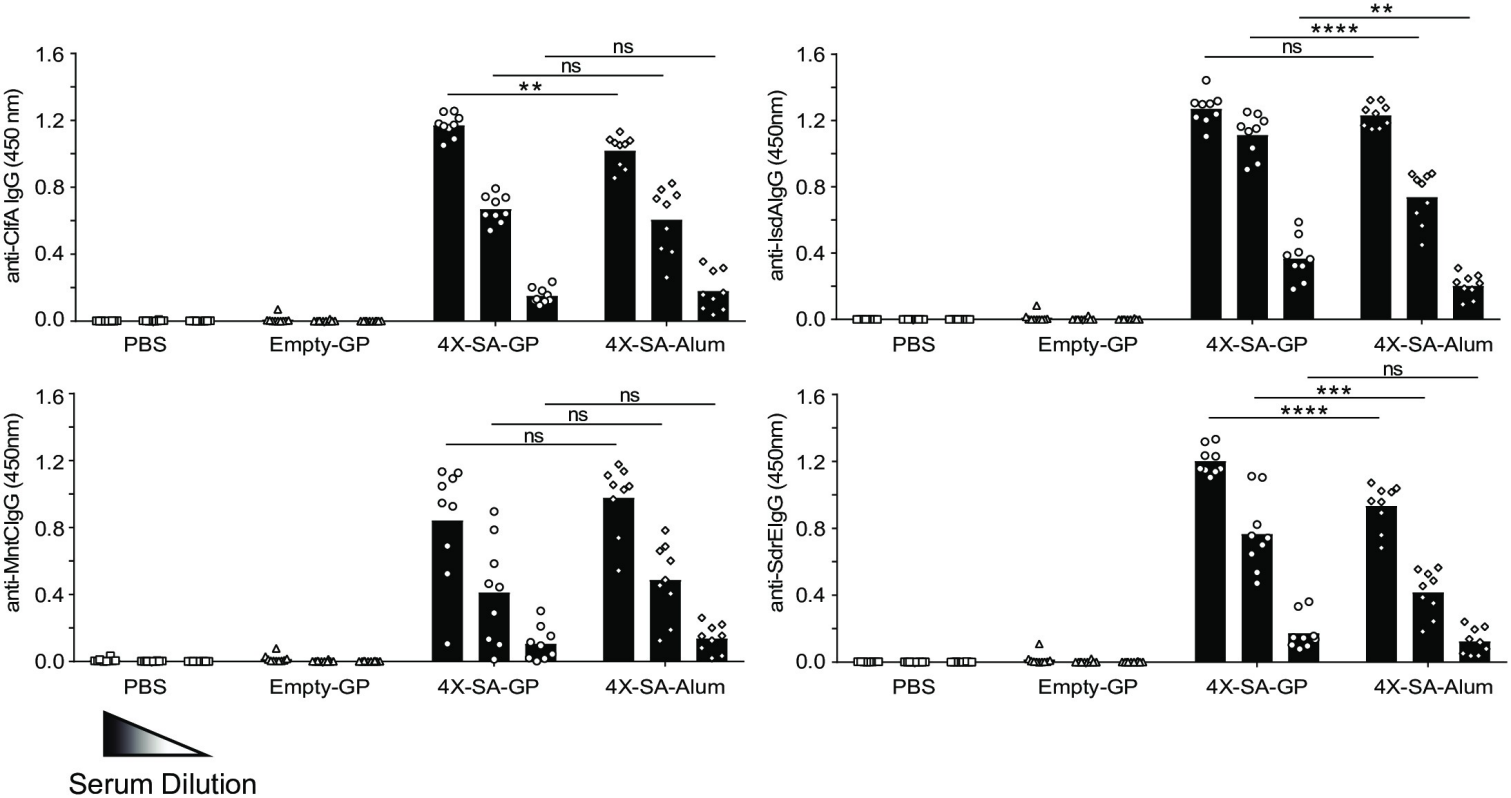
4X-SA-GP induced antibody responses are similar to those induced by the common adjuvant alum. Wild-type female mice were immunized once a week for 3 weeks with PBS (n = 10), Empty-GPs (n = 10), 4X-SA-GP (n = 9) or the same amount of *S*. *aureus* antigens mixed with alum (n = 9). Serum was collected from each group 2 weeks after the third and final immunization. The serum was diluted 10X 3 times starting at 1:1000 and was then tested for total IgG antibodies specific for each of the 4 *S*. *aureus* proteins included in the vaccine by ELISA. The read-out of the assay is the optical density (OD) at 450 nm for each serum sample. Data are representative of three experiments and were analyzed using ANOVA and t test at each dilution. **p<0.01, ***p<0.001, ****p<0.0001; ns, not significant.

We also directly compared antibody production induced by 4X-SA-GP to antibodies produced when the widely used antibody-promoting adjuvant alum (4X-SA-Alum) was used. Mice were immunized three times as above, and an additional cohort of mice was immunized by the same route and schedule with matching amounts of the four *S*. *aureus* antigens mixed with alum. The 4X-SA-GP vaccine induced antibodies comparably, if not better than, the alum adjuvanted approach (Fig 6). Taken together, the data indicate that vaccination of mice three times with 4X-SA-GP promotes protection in mice against *S*. *aureus* infection and induces strong antigen-specific CD4^+^ T cell and antibody responses.

### Transferred antibodies do not elicit protection and CD4^+^ T cells are needed for the efficacy of the 4X-SA-GP vaccine in mice

**Fig 7:**
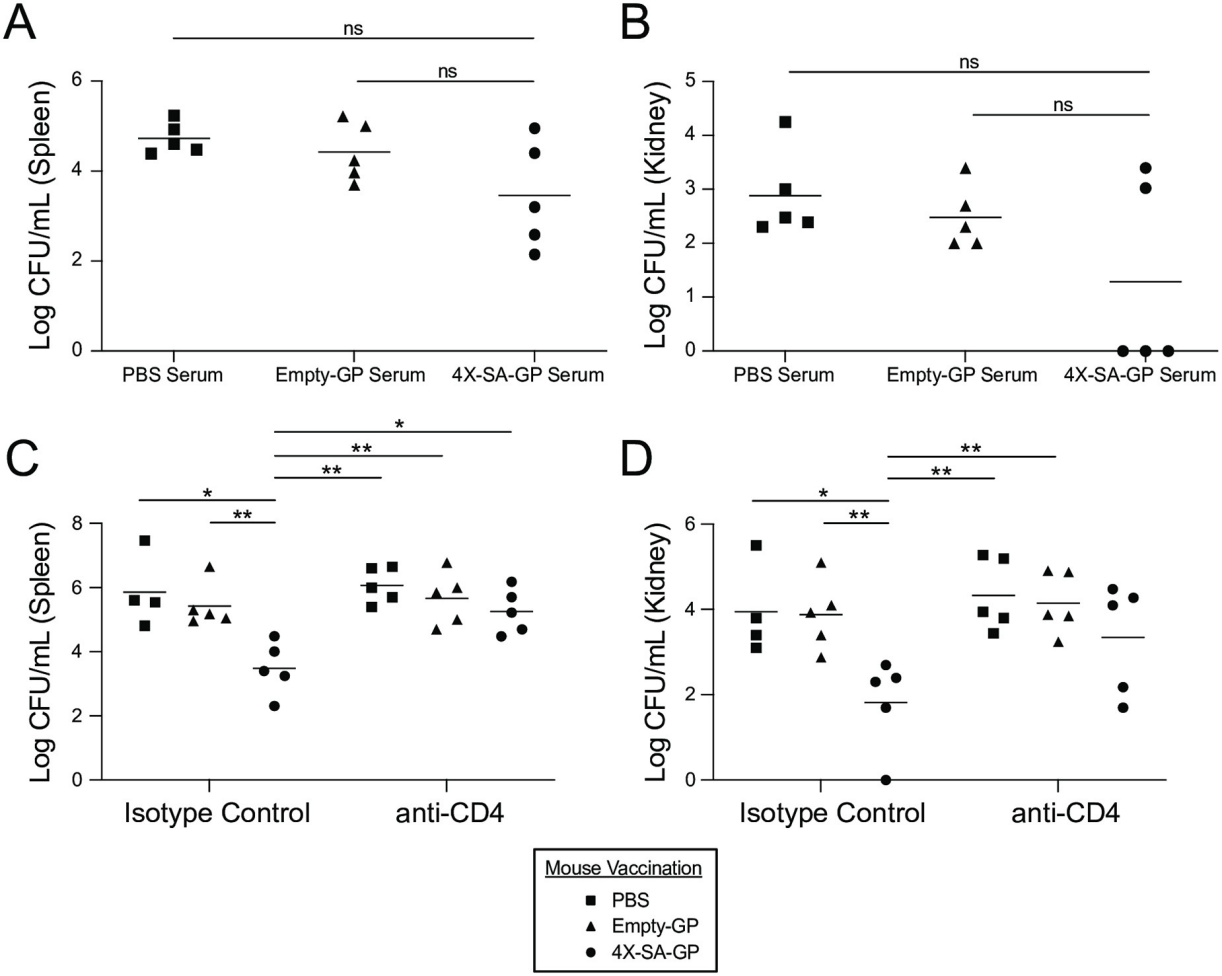
Antibodies alone are not sufficient to provide 4X-SA-GP-induced protection against systemic *S*. *aureus* infection, while CD4^+^ T cells are indispensable. (A and B) Wild-type female mice were immunized once a week for 3 weeks with PBS, Empty-GP, or 4X-SA-GP and serum was collected from each group of mice 2 weeks after the final vaccination. The serum from each group of mice was pooled and injected i.p. into wild-type female naïve recipient mice (n = 5 mice/group, PBS serum, Empty-GP serum, 4X-SA-GP serum). The recipient mice were infected i.p. with 2x10^7^ CFUs of *S*. *aureus* (LAC USA300) the following day. Bacteria from the spleen (A) and kidneys (B) were recovered after 24 hours and CFUs were enumerated. (C and D) Wild-type female mice were immunized once a week for 3 weeks with PBS (n = 9), Empty-GPs (n = 10), or 4X-SA-GP (n = 10). Four weeks after the final vaccination 4–5 mice per vaccination group were treated i.p. with anti-CD4^+^ antibody or the corresponding isotype control antibody on day -1 and day 0. On day 0, all groups of the mice were infected i.p. with 2x10^7^ CFUs of *S*. *aureus* (LAC USA300). Bacteria from the spleen (C) and kidneys (D) were recovered after 24 hours after the *S*. *aureus* infection and CFUs were enumerated. Each data point represents an individual mouse. Data from the serum transfer is representative of two experiments. Data from the CD4^+^ T cell depletion is from a single experiment. Data analysis was performed using Kruskal-Wallis test ((A) = 0.18, (B) = 0.46, (C) = 0.0.02, (D) = 0.05) and Mann-Whitney U test. *p<0.05, **p<0.01; ns, not significant.

A successful *S*. *aureus* vaccine should most likely elicit both antibody and CD4^+^ T cell responses [7, 14]. To test whether the antibodies produced in response to 4X-SA-GP are enough to provide protection on their own, we performed a serum transfer. We collected serum from mice vaccinated with 4X-SA-GP, empty-GPs, or PBS and transferred the serum to naïve recipient mice via i.p. injection. The following day, we infected the recipient mice with *S*. *aureus* and assessed spleen and kidney CFUs after 24 hours. There were no differences in the bacterial burdens in the spleen (Fig 7A) or kidneys (Fig 7B) of mice that received the 4X-SA-GP serum compared to mice that received PBS or empty-GP serum. Mice that were given the 4X-SA-GP serum had lower CFU count averages in the spleen and kidneys (Fig 7A and 7B) in comparison to the PBS and empty-GP serum groups suggesting that the antigen-specific antibodies from the 4X-SA-GP serum may have a modest protective effect, though not enough to formally provide significant protection.

To evaluate the role of antigen-specific CD4^+^ T cells in vaccine-induced protection, we depleted CD4^+^ T cells prior to infection. Vaccinated mice from each group (PBS, empty-GP, 4X-SA-GP) received an i.p. injection of either anti-CD4^+^ antibody or the corresponding isotype control antibody four weeks post-vaccination. To ensure depletion of the CD4^+^ T cells, we measured CD4^+^ T cell percentages prior to infection in peripheral blood mononuclear cells (PBMCs) isolated from each group of mice and post-infection in the MLNs by flow cytometry (S5 Fig). Upon infection with *S*. *aureus*, we observed that depletion of T cells blocked the protection offered by the 4X-SA-GP vaccine (Fig 7C and 7D).

### Mice are protected from systemic *S*. *aureus* infection eight weeks after vaccination with 4X-SA-GP

**Fig 8:**
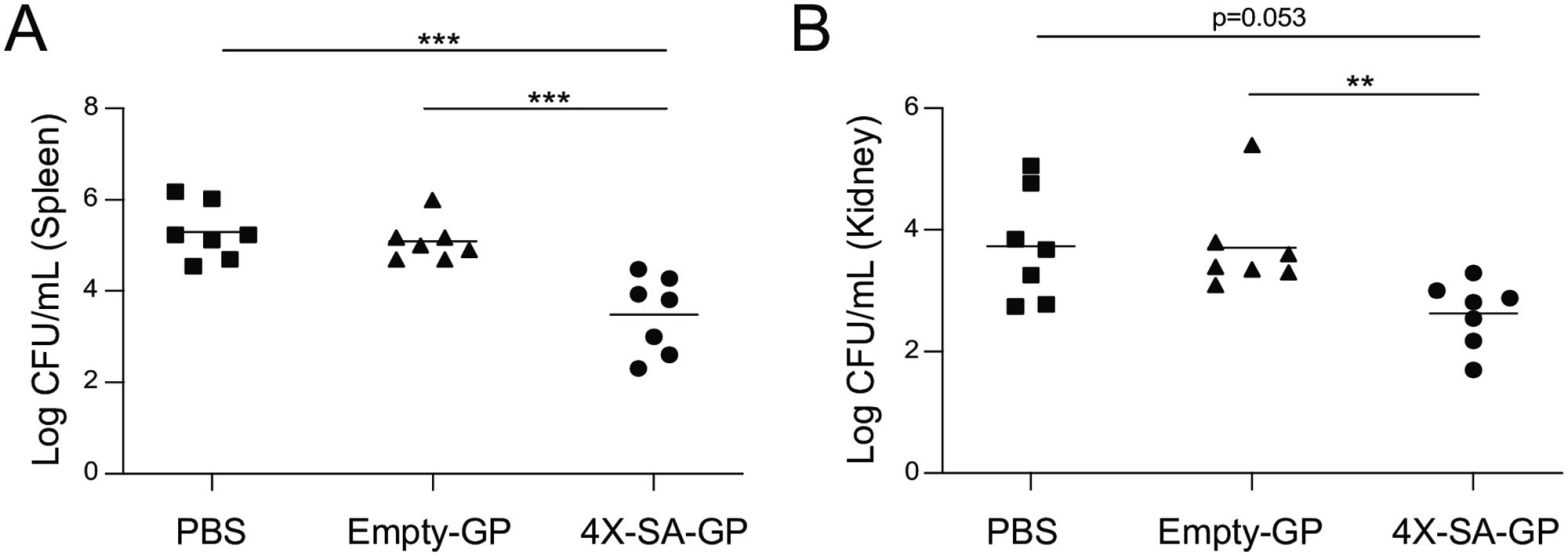
Protective immunity from 4X-SA-GP against *S*. *aureus* infection is long lasting. (A and B) Wild-type female mice were immunized once a week for 3 weeks with PBS (n = 7), Empty-GPs (n = 7), or 4X-SA-GP (n = 7). Eight weeks after the final vaccination, mice were infected i.p. with 2x10^7^ CFUs of *S*. *aureus* (LAC USA300). Bacteria from the spleen (A) and kidneys (B) were recovered after 24 hours and CFUs were enumerated. Each data point represents an individual mouse. Data analysis was performed using Kruskal-Wallis test ((A) = 0.0001 (B) = 0.008, and Mann-Whitney U test. **p<0.01, ***p<0.005; ns, not significant. Data are from a single experiment.

We next sought to establish if the protective immunity induced by 4X-SA-GP vaccination could provide protection after a time period longer than four weeks. We vaccinated animals three times as before, but we rested them for eight weeks prior to infection. 4X-SA-GP vaccinated mice were still significantly protected after 8 weeks (Fig 8). Intriguingly, at this later time point, the protection provided by the empty-GP vaccinated mice appears to have faded entirely (Fig 8A and 8B). Moreover, eight weeks after the final vaccination, the relative antibody levels of IgG1 and IgG2c subclasses specific for each protein were strong, detectable and often comparable to the levels seen in serum collected from mice two weeks post-vaccination (S6A and S6B Fig). These results suggest that vaccination of mice with three doses of 4X-SA-GP induces long-lasting protective immunity against *S*. *aureus* with antigen-specific adaptive cellular and humoral immune responses.

## Discussion

*Staphylococcus aureus* is the most frequent cause of infections in the United States. Antibiotic resistant *S*. *aureus* strains, such as Methicillin-Resistant *S*. *aureus* (MRSA), have rapidly emerged worldwide, infecting immunocompromised and healthy individuals alike, making it a major public health threat [8]. Vaccines aimed at targeting *S*. *aureus* have failed in clinical trials [9] and the reason for this lack of success remains unknown. As this pathogen continues to rapidly spread on a global scale, it is vital that new approaches towards a *S*. *aureus* vaccine emerge. The observation that conditions that predispose a person to *S*. *aureus* infections often also predispose to fungal infections suggests that the elements of immunity that are effective against *S*. *aureus* share much in common with the elements of immunity that promote defense against fungal infections. We therefore hypothesized that a vaccine based on activating antifungal innate immune responses in the context of delivery of *S*. *aureus* antigens might be productive. In this study, we developed and tested in mice a vaccine candidate consisting of fungal β-glucan particles loaded with four *S*. *aureus* protein antigens (4X-SA-GP).

Of the four *S*. *aureus* protein antigens we used, ClfA and MntC have and are being used (together with capsular polysaccharides) in vaccines that have gone on to clinical trials. In this case, the vaccine employs a non-toxic mutant of diphtheria toxin cross-reacting material 197 (CRM197) as an adjuvant, but it has so far not proven effective at preventing infection. The vaccine generates antibodies to ClfA and MntC that would appear to be effective in vitro [58]. A variety of other antigens have moved to clinical trials as well, but none yet have proven effective [59]. A working hypothesis is that a successful vaccine will need to generate a protective T cell response instead of or in addition to an antibody response [60].

Vaccinating mice with 3 doses of 4X-SA-GP induced long lasting (at least 8 weeks) cellular and humoral antigen specific immune responses. Antigen-specific T cell responses were polarized towards Th1 and Th17 as would be anticipated for the adjuvant activity of β-glucan, and these T cell responses were essential for vaccine-induced protection from systemic infection with *S*. *aureus*. The vaccine also induced strong IgG1 and IgG2c antibody responses to the *S*. *aureus* proteins, although it was not clear that these antibody responses were sufficient to mediate protection from infection.

Four weeks after completing the vaccination, animals retained some variable non-specific enhanced resistance to *S*. *aureus* infection that was induced by β-glucan particles having no *S*. *aureus* antigens. This immunity wore off after 8 weeks, but the antigen specific immunity induced by antigen-loaded particles persisted. It is therefore possible that the β-glucan particles alone elicit some short-lived non-antigen-specific inflammatory immunity by which innate immune cells show enhanced responsiveness. Future studies could be designed to characterize the cellular and molecular signatures associated with this temporary protection, but the antigen-specific responses are longer-lived.

Some years ago, a group of investigators working on development of a *Candida albicans* vaccine discovered that their vaccine provided cross-protection from *S*. *aureus* [61]. The vaccine employs recombinant *Candida* agglutinin-like sequence 3 (Als3) adhesin/invasin as the antigen and is mixed with alum as an adjuvant. While the vaccine induces good antibody production against Als3 that cross react with *S*. *aureus* cell walls, protection in mouse models of infection required T cell mediated immunity (especially Th1 and Th17 polarized responses), not B cell immunity [62, 63]. Polarization of CD4^+^ T cells to both the Th1 and Th17 subsets in response to the 4X-SA-GP may make this approach more desirable compared to vaccines that polarize T cells to only one main subset. Th17 cells appear to play a major role in eliciting protection for skin, respiratory, and mucocutaneous infections [64, 65], while Th1 cells play a protective role in bloodstream infection [62]. Therefore, differentiation of CD4^+^ T cells to both these subsets by 4X-SA-GP immunization may help ensure that an effective T cell-mediated downstream response will occur upon infection. Previous studies have indicated that *S*. *aureus*-specific T cells that stimulate phagocyte recruitment and activation through the production of the proinflammatory cytokines IFNγ and/or IL-17 are important for immunity to *S*. *aureus* [61, 66].

The GP vaccine platform has been previously investigated primarily in the context of developing fungal vaccines [28, 67] due to the lack of effective vaccines for fungal infections. Fungi resist lysis by the complement system, but the deposition of iC3b on a fungus prompts opsonization and phagocytosis [68]. In many cases, antibodies are not protective against fungal infections [69], and some vaccines are now focusing on producing cellular immune responses as well [28] given that T cells are major players in fighting fungal infections. Studies focusing on candidiasis and aspergillosis show that Th1 and Th17 responses are more important than generation of neutralizing antibodies [70]. Vaccination of mice with glucan particles loaded with *Cryptococcus*-derived alkaline extracts protected animals from a lethal challenge with *Cryptococcus neoformans* and *Cryptococcus gattii* [28]. Glucan particles alone as a vaccine also afforded mice protection against *Aspergillus fumigatus* infection [67]. Thus, studies evaluating GPs as vaccine platforms for inducing antifungal immunity are farther along than evaluation of GPs as a vaccine for *S*. *aureus*, and they demonstrate the potential GPs have as a vaccine to induce protective cellular immunity. The similarities between the immune responses needed for antifungal and anti-staphylococcal immunity suggest that similar vaccination strategies might be effective, and further studies will be required to more completely optimize a GP-based *S*. *aureus* vaccine.

## Materials and methods

### Ethics statement

This study was performed under strict accordance with the recommendations in the Guide for the Care and Use of Laboratory Animals. This protocol was approved by the institutional animal use and care committee of the Cedars-Sinai Medical Center (IACUC 7174 and 8836).

### Microbes

For all *S*. *aureus* infection experiments, the dominant CA-MRSA strain in the United States, USA300, specifically the Los Angeles County clone (LAC) was used [71]. *S*. *aureus* LAC was routinely cultured in Tryptic Soy Broth. Mid-log phase bacteria sub-cultured from overnight cultures were used for experiments. Bacteria were washed in PBS twice prior to use and inocula were confirmed by CFU determination on blood agar plates. Heat-killed *S*. *aureus* was prepared by incubating a washed overnight culture of *S*. *aureus* for 45 minutes at 90°C. The bacteria were then washed to remove secreted proteins. The sterility of killed bacteria was confirmed by enumeration of CFU on an agar plate.

### Mice

C57BL/6 mice were purchased from Jackson Laboratories and Rag2/OT-II transgenic mice were purchased from Taconic. OT-II TCR transgenic mice and Dectin-1 knockout mice were bred and housed under specific pathogen-free conditions in the Cedars-Sinai Medical Center animal facility. Aged-matched female mice aged 8–12 weeks were used for *in vitro* and *in vivo* experiments. Due to sex-related differences in susceptibility to *S*. *aureus* [72], only female mice were used in this study.

### Primary Cells

Bone marrow-derived dendritic cells (BMDCs) from C57BL/6 and Dectin-1 knockout mice were grown as previously described [73] in RPMI (Corning) supplemented with 10% fetal bovine serum (FBS) and 10 ng/mL recombinant murine granulocyte-monocyte colony-stimulating factor (GM-CSF) (Peprotech). Between days 7–10, the differentiated cells were used for *in vitro* stimulation assays. CD4^+^ T cells were isolated from pooled spleens and lymph nodes of 8-12-week-old female OT-II or Rag2/OT-II mice using an EasySep mouse naïve CD4^+^ T cell Isolation kit (STEMCELL Technologies).

### BMDC stimulation

BMDCs were plated at 5x10^5^ cells/well in 24-well TC plates (for flow cytometry analysis) or 2x10^5^ cells/well in 96 well round-bottom TC plates (for T cell co-culture). The following day after re-plating, BMDCs were stimulated for 24 hours with GP-OVA (10 particles/cell), 4X-SA-GP (10 particles/cell), or 100 ng/mL LPS (Invivogen). For cytokine analysis of BMDCs, supernatants were harvested 24 hours after stimulation, and for analysis of maturation markers, BMDCs were lifted after overnight stimulation with PBS containing 2mM EDTA, followed by immunofluorescent staining and flow cytometry.

### ELISA and flow cytometry

Enzyme linked immunosorbent assays (ELISA) were performed according to the manufacturer’s instructions for IL-6, TNFα, IL-17A, and IFNγ (BioLegend). For antibody analysis in the serum of mice, ELISA plates (Corning Costar) were coated with 5 μg per well of rClfA, rIsdA, rMntC, rSdrE at 4°C overnight. The plates were blocked with ELISA assay diluent (BioLegend) followed by incubation with the sera from vaccinated mice at dilutions of 1:1000, 1:10,000, and 1:100,000 in the assay diluent. The plates were then incubated with biotin-conjugated anti-mouse IgG1 (BioLegend) followed by streptavidin-conjugated anti-mouse IgG-HRP (BioLegend). For IgG2c, sera were incubated with anti-mouse IgG2c-HRP (SouthernBiotec). The plates were then incubated with TMB substrate solution (BD OptEIA) and the reactions were stopped with 2N H_2_SO_4_ and the plates were read at 450 nm.

Fluorophore-conjugated anti-mouse antibodies directed against the following molecules were used to stain cells: CD11b (M1/70), CD11c (N418), Ly6C (HK1.4), Ly6G (1A8), F4/80 (BM8), CD19 (1D3/CD19), I-A/I-E (M5/114.15.2, CD80 (16-10A1), CD86 (GL-1), IL-17A (TC11-8H10.1), IFNγ (XMG1.2), CD3 (145-2C11), TCRβ (H57-597), CD4 (GK1.5), TCR Vβ5.1/5.2 (MR9-4), TCR Vα2 (B20.1) (all from BioLegend). All samples were prestained with anti-CD16/CD32 (eBioscience) to block FC receptors, and Zombie fixable viability dye (BioLegend) to identify dead cells. For intracellular cytokine staining, cells were stimulated for 4 hours with Cell Activation Cocktail (BioLegend) in the presence of GolgiStop (BD Biosciences) for the last 3 hours of stimulation. Following the addition of Fc-block, viability dye and surface staining, intracellular cytokine staining was performed using the cytofix/cytoperm staining kit (BD Biosciences) according to the manufacturer’s instructions. Samples were acquired with a LSRII (BD Biosciences) and data were analyzed with FlowJo software (Tree star).

### Western Blotting

For assessing ovalbumin or the recombinant proteins in the glucan particles, 2x10^6^ particles were loaded onto a gradient gel and subjected to gel electrophoresis using the Novex NuPAGE Gel electrophoresis system (Invitrogen), and proteins were transferred onto a polyvinylidene difluoride (PVDF) membrane (Millipore). GP-OVA blots were probed with an anti-ovalbumin antibody (anti-albumin, Calbiochem), followed by an HRP-conjugated secondary antibody (Jackson ImmunoResearch). Blots were incubated with ECL substrate (Pierce) and then exposed to X-ray film. 4X-SA-GP blots were probed with an anti-6xHN antibody (Clontech Laboratories) to detect the polyhistidine tag on the N-terminus of each of the recombinant proteins followed by infrared dye-conjugated secondary antibodies (LI-COR). Bands were visualized on an Odyssey imaging system (LI-COR).

### DC: OT-II T cell co-culture

BMDCs were plated in 96 well round-bottom TC plates at 2x10^5^ cells/well and were stimulated overnight with GP-No OVA or GP-OVA (10 particles/cell). The following day, the BMDCs were washed thoroughly and CFSE-labeled [74] naïve OT-II CD4^+^ T cells were added at a 1:5 ratio (2x10^5^ DC: 1x10^6^ T cells) and cells were cultured with RPMI supplemented with 10% FBS, 1 mM sodium pyruvate and 55 μM 2-mercaptoethanol. Following 4–5 days of co-culture, supernatants were harvested for cytokine analysis by ELISA and T cells were restimulated with Cell Activation Cocktail (BioLegend) and Golgistop (BD Biosciences) and stained intracellularly with fluorophore-conjugated antibodies against IL-17A and IFNγ.

### Antigen-loaded glucan particles

β-glucan particles were prepared by treating *Saccharomyces cerevisiae* zymosan A (Sigma) with a hot-alkali treatment to remove TLR agonists as previously described [75]. Briefly, a suspension of zymosan particles were initially prepared and the particles were then boiled in 10 M NaOH for 1 hour followed by 5 washes in sterile PBS. The glucan particles were then dried in a Savant SpeedVac vacuum concentrator. Dried glucan particles were loaded with various concentrations of ovalbumin (OVA) or the recombinant *S*. *aureus* proteins (with or without TRITC or FITC-labeling) as previously described [29, 51]. Glucan particles (GPs) were kept as frozen aliquots and were sonicated in an ultrasonic water bath for 15 minutes prior to their *in vitro* or *in vivo* use. Empty-GPs were prepared the same as the loaded-GPs minus the addition of any protein antigen.

### Cloning and purification of recombinant proteins

Coding sequences for ClfA, IsdA, MntC, and SdrE were PCR amplified without the signal peptide or sorting signal (see Table 1 for a list of primer sequences) as previously described [41]. PCR products were cloned into pET6xHN Expression Vector using the In-Fusion Cloning Kit (Clontech Laboratories) to express recombinant proteins that have a N-terminal 6xHN fusion tag. The recombinant proteins were purified from bacterial-clarified lysates by using His60 Ni Superflow Resin (Ni-IDA resin), His60 Ni Buffers and gravity columns (ClonTech Laboratories) per the manufacturer’s instructions. Purified proteins underwent endotoxin removal via endotoxin removal spin columns (Pierce), were concentrated with Amicon Ultra centrifugal filter units (Millipore), and the final protein concentration was determined by BCA protein assay (Pierce).

**Table 1:** List of primer sequences for In-Fusion Cloning, 5’ to 3’.

Primer	Sequence 5’ to 3’
ClfA forward	TAAGGCCTCTGTCGACAGTGAAAATAGTGTTACGCAA
ClfA reverse	CAGAATTCGCAAGCTTTGTATCTGGTAATGGTTCTTT
IsdA forward	TAAGGCCTCTGTCGACGCAACAGAAGCTACGAACG
IsdA reverse	CAGAATTCGCAAGCTTAGTTTTTGGTAATTCTTTAGCT
MntC forward	TAAGGCCTCTGTCGACAGTGATAAGTCAAATGGCAAA
MntC reverse	CAGAATTCGCAAGCTTTTATTTCATGCTTCCGTGTAC
SdrE forward	TAAGGCCTCTGTCGACGCTGAAAACACTAGTACAGA
SdrE reverse	CAGAATTCGCAAGCTTTGTTTCTGGTAATGCTTTTGC

### Intraperitoneal injections of FITC-labeled 4X-SA-GP

Mice were injected intraperitoneally with 5x10^7^ FITC-labeled or unlabeled 4X-SA-GP and allowed to rest for 24, 48, or 72 hours before being euthanized. The peritoneal cavity was lavaged with 10 ml of cold PBS with 2 mM EDTA. The cells there then counted and stained for the surface markers CD11b, CD11c, Ly6C, Ly6G, F4/80, and CD19 and analyzed by flow cytometry. The cell number per mL (gated first on FITC^+^ cells) was determined via CountBright Absolute Counting Beads (Invitrogen).

### *Ex vivo* stimulation of splenocytes

Spleens were isolated 5 days after the final vaccination of mice with PBS, empty-GP, or 4X-SA-GP. Red blood cells were lysed and cells were resuspended in RPMI supplemented with 10% FBS, 1 mM sodium pyruvate, and 55 μM 2-mercaptoethanol and plated at 3x10^6^ cells/well. For specific stimulation, cells were cultured with a mixture of the 4 recombinant proteins (10 μg/protein per mL) or heat-killed *S*. *aureus* at an MOI of 5. Supernatants were collected after 72 hours for analysis of cytokines by ELISA. For intracellular cytokine analysis, the cells were stimulated with Cell Activation Cocktail (BioLegend) for 4 hours and Golgistop (BD Biosciences) for the last 3 hours of stimulation. Intracellular cytokines (IL-17A and IFNγ) were then analyzed by flow cytometry.

### Vaccination and infection model

Single vaccination model: Mice were vaccinated once with PBS as a control, empty-GPs, or 4X-SA-GP (5x10^7^ GPs). Mice were rested for 5 days before the spleen and MLNs were harvested for the T cell restimulation assay. For the infection model, mice were rested for 4 weeks after the one vaccination and were then inoculated with 2x10^7^ CFU of *S*. *aureus* (LAC USA300) via i.p. injection. Mice were euthanized 24 hours after the infection and spleen and kidneys were harvested, homogenized, and plated on blood agar plates. CFUs were enumerated after overnight incubation at 37°C.

Triple vaccination model: Mice were vaccinated once a week for 3 weeks with PBS as a control, empty-GPs, or 4X-SA-GP (5x10^7^ GPs/dose). For comparison to alum, mice were immunized with payload-matched amounts of *S*. *aureus* antigens (50 μg/injection) mixed with alum (1 mg/injection). The quantities of the glucan particle-loaded recombinant proteins were estimated by knowing the amount of protein added to a particle preparation and subtracting the amount left over in supernatants. Mice were rested for 5 days before the spleen and MLNs were harvested for the T cell restimulation assay. For the infection model, mice were rested for 4 or 8 weeks (where indicated) after the final vaccination and were then inoculated with 2x10^7^ CFU of *S*. *aureus* (LAC USA300). Mice were euthanized 24 hours after the infection and spleen and kidneys were harvested, homogenized, and plated on blood agar plates. CFUs were enumerated after overnight incubation at 37°C.

### Serum transfer

Mice were vaccinated once a week for 3 weeks with PBS as a control, empty-GPs, or 4X-SA-GP (5x10^7^ GPs/dose). Two weeks after the final vaccination, serum was collected from all of the mice and pooled according to the vaccination group. Recipient mice received an i.p. injection of 150 μL of the pooled serum on day -1 and were then inoculated with 2x10^7^ CFU of *S*. *aureus* (LAC USA300) via i.p. injection the following morning on day 0. Mice were euthanized 24 hours after the infection and spleen and kidneys were harvested, homogenized, and plated on blood agar plates. CFUs were enumerated after overnight incubation at 37°C.

### CD4^+^ T cell depletion

Mice were vaccinated once a week for 3 weeks with PBS as a control, empty-GPs, or 4X-SA-GP (5x10^7^ GPs/dose). Mice were rested for 4 weeks and then received an i.p. injection with an anti-CD4^+^ antibody (clone Gk1.5, BioXCell) 300 μg in 500 μl PBS on day -1 and 100 μg in 500 μl PBS on day 0 as previously described [76]. Control mice were treated with an i.p. injection of the corresponding isotype control antibody (BioXCell) at the same dosage and volume. On day 0, all mice were then inoculated with 2x10^7^ CFU of *S*. *aureus* (LAC USA300) via i.p. injection. Mice were euthanized 24 hours after the infection and spleen and kidneys were harvested, homogenized, and plated on blood agar plates. CFUs were enumerated after overnight incubation at 37°C. PBMCs were isolated from the blood of mice and pooled from all groups of mice on day 0 prior to infection and were stained with the surface markers CD3, CD4, and TCRβ and analyzed by flow cytometry to assess the efficacy of the CD4^+^ T cell depletion. MLNs were harvested on day 1, after the infection, and the isolated cells were stained for the surface markers CD3, CD4, and TCRβ and analyzed via flow cytometry to assess that the CD4^+^ T cells were still depleted post-infection.

### Statistical analysis

Statistical analysis was determined by Student’s t test, two-way ANOVA with the Tukey multiple comparisons post-hoc test (denoted ANOVA in figure legends), or Mann-Whitney U test using GraphPad Prism software. All statistical details of experiments such as the number of replicates can be found in the figure legends. Where present, error bars indicate the mean +SD. p values less than 0.05 are considered significant.

### Schematics

S1A Fig schematic was created using BioRender.com.

## Supporting information

S1 Fig Generation of 4X-SA-GP, a *S*. *aureus* vaccine strategy utilizing glucan particles. (A) Schematic for the generation of 4X-SA-GP. Glucan particles were loaded with the purified, his-tagged, recombinant proteins: rClfA, rIsdA, rMntC, and rSdrE. (B) Coomassie stained SDS-PAGE gel of the 4 purified recombinant proteins: Lane 1, rClfA (98.3 kDa), Lane 2, rMntC (36.2 kDa), Lane 3, rIsdA (34.1 kDa), Lane 4, rSdrE (118.6 kDa). The approximate molecular weight of each protein is shown in parentheses. All lanes contain approximately 10 μg of protein. (C). Western blot analysis of 4X-SA-GP using an anti-6xHN antibody to probe for the his-tagged proteins within the GPs. 2x10^7^ 4X-SA-GPs were loaded into the lane.
(TIF)

S2 Fig GP-OVA promotes proliferation and polarization of naïve CD4^+^ T cells to the Th1 and Th17 subsets *in vitro*. (A-G) BMDCs stimulated with GP-No OVA or GP-OVA (loaded with various concentrations of OVA) were used to activate naïve CFSE-labeled OT-II CD4^+^ T cells under non-polarizing conditions. (A) Representative flow cytometry plots show the degree of Th1 (IFNγ^+^) and Th17 (IL-17^+^) T cell differentiation under each condition of BMDC activation. (B) Representative flow cytometry histograms demonstrate the degree of T cell polarization corresponding to the data above with the pink histogram from the co-culture with no stimulation used as a reference point for the percentage of proliferation. (C-D) Bar graphs quantifying the percentage of IL-17^+^ (C) and IFNγ^+^ (D) OT-II cells and the percentage of CFSE proliferation (E). (F-G) Supernatants from the co-culture were collected on day 5 and analyzed for production of IL-17 (F) and IFNγ (G) by ELISA. Flow cytometry percentages and ELISA data and their standard deviation are representative of two replicates/condition. Data are representative of two experiments.
(TIF)

S3 Fig One immunization with 4X-SA-GP promotes less than optimal antigen-specific CD4^+^ T cell responses. (A-E) Wild-type female mice were immunized one time with PBS (n = 5), Empty-GPs (n = 5), or 4X-SA-GP (n = 5). Five days after the vaccination, splenocytes from each mouse were stimulated with a mixture of all 4 *S*. *aureus* purified recombinant proteins (proteins) and heat-killed *S*. *aureus* (HK-SA) for 3 days. (No stim., no stimulation). After 3 days, the splenocytes were stimulated with phorbol myristate acetate (PMA) and ionomycin and then analyzed by flow cytometry after intracellular cytokine staining. Representative flow cytometry plots demonstrate the degree of IL-17 and IFNγ production from CD4^+^ T cells (gated on TCRβ^+^CD4^+^) in response to the stimuli (proteins, HK-SA) from the spleen (A). (B and D) Bar graphs show the frequency of IL-17 (B) and IFNγ positive cells in the spleen from the mice. Each data point represents an individual mouse in all of the bar graphs. (C and E) Supernatants were harvested at day 3 from the splenocyte (A) stimulation and the cytokines IL-17 (C) and IFNγ (E) were determined by ELISA. Data analysis was performed using ANOVA. *p<0.05, **p<0.01, ***p<0.005, ****p<0.0001. Data are representative of a single experiment.
(TIF)

S4 Fig Mice receiving one 4X-SA-GP vaccination do not develop robust antibody responses. (A-B) Serum was collected from each group of vaccinated mice 2 weeks after one immunization with PBS, Empty-GP, or 4X-SA-GP (n = 5 mice per group). The serum was diluted 3 times at 1:1000, 1:10,000 and 1;100,000 and was then tested for antibodies specific for each of the 4 *S*. *aureus* proteins encapsulated in 4X-SA-GP by ELISA. The subclasses IgG1 (A) and IgG2c (B) were analyzed for specificity towards rClfA, rIsdA, rMntC, and SdrE. The read-out of the assay is the optical density (OD) at 450 nm for each serum sample. Each data point represents an individual mouse. Data analysis was performed using ANOVA. *p<0.05, **p<0.01, ***p<0.001, ****p<0.0001. Data are representative of a single experiment.
(TIF)

S5 Fig Flow cytometry analysis of CD4+ T cell percentages after treatment with depletion antibody. (A-B) Wild-type female mice were immunized once a week for 3 weeks with PBS (n = 9), Empty-GPs (n = 10), or 4X-SA-GP (n = 10). Four weeks after the final vaccination 4–5 mice per vaccination group were treated i.p. with anti-CD4^+^ antibody or the corresponding isotype control antibody on day -1 and day 0. On day 0, all groups of the mice were infected i.p. with 2x10^7^ CFUs of *S*. *aureus* (LAC USA300). (A) flow cytometry plots demonstrating the degree of CD4^+^ T cell depletion from pooled peripheral blood mononuclear cells (PBMCs) from each vaccination group of mice on day 0 before the mice were infected with *S*. *aureus*. (B) flow cytometry plots demonstrating the degree of CD4^+^ T cell depletion from pooled MLNs from each vaccination group of mice 24 hours after the mice were infected with *S*. *aureus* (day 1). Cells from (A) and (B) were gated on CD3^+^ cells.
(TIF)

S6 Fig 4X-SA-GP vaccination induces long-term antibody responses in mice. (A-B) Two sets of wild-type female mice were immunized once a week for 3 weeks with PBS (n = 5), Empty-GPs (n = 5), or 4X-SA-GP (n = 5). Serum was collected from one set of mice (PBS, Empty-GP, 4X-SA-GP; n = 5 mice/group) 2 weeks after the final vaccination and 8 weeks after the final immunization for the other set of mice (PBS, Empty-GP, 4X-SA-GP; n = 5 mice/group). The serum was diluted 3 times at 1:1000, 1:10,000 and 1;100,000 and was then tested for antibodies specific for each of the 4 *S*. *aureus* proteins encapsulated in 4X-SA-GP by ELISA. The subclasses IgG1(A) and IgG2c (B) were analyzed for specificity towards rClfA, rIsdA, rMntC, and rSdrE. The read-out of the assay is the optical density (OD) at 450 nm for each serum sample. Each data point represents an individual mouse. Data analysis was performed using ANOVA. *p<0.05, **p<0.005. Data are representative of at least two experiments for serum at two weeks and a single experiment for serum at 8 weeks.
(TIF)
