# Supplementary figures and images for: Harnessing antifungal immunity in pursuit of a Staphylococcus aureus vaccine strategy
Source: PLoS Pathog. 2020 Aug 20;16(8):e1008733. doi: 10.1371/journal.ppat.1008733 (PMC7446838; doi:10.1371/journal.ppat.1008733)

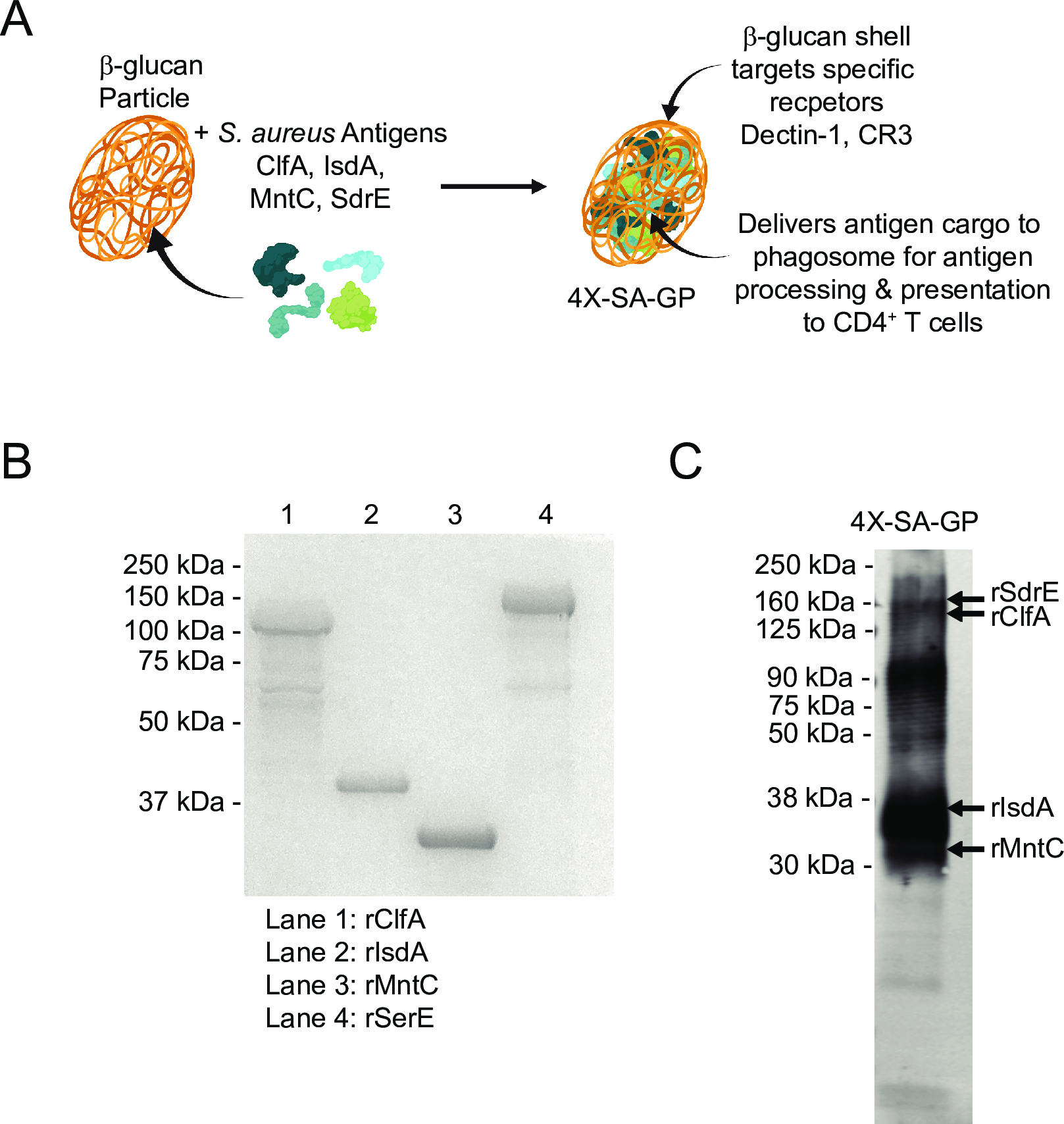

Supplement: S1 Fig — (A) Schematic for the generation of 4X-SA-GP. Glucan particles were loaded with the purified, his-tagged, recombinant proteins: rClfA, rIsdA, rMntC, and rSdrE. (B) Coomassie stained SDS-PAGE gel of the 4 purified recombinant proteins: Lane 1, rClfA (98.3 kDa), Lane 2, rMntC (36.2 kDa), Lane 3, rIsdA (34.1 kDa), Lane 4, rSdrE (118.6 kDa). The approximate molecular weight of each protein is shown in parentheses. All lanes contain approximately 10 μg of protein. (C). Western blot analysis of 4X-SA-GP using an anti-6xHN antibody to probe for the his-tagged proteins within the GPs. 2x10^7^ 4X-SA-GPs were loaded into the lane. (TIF) [file ppat.1008733.s001.tif]

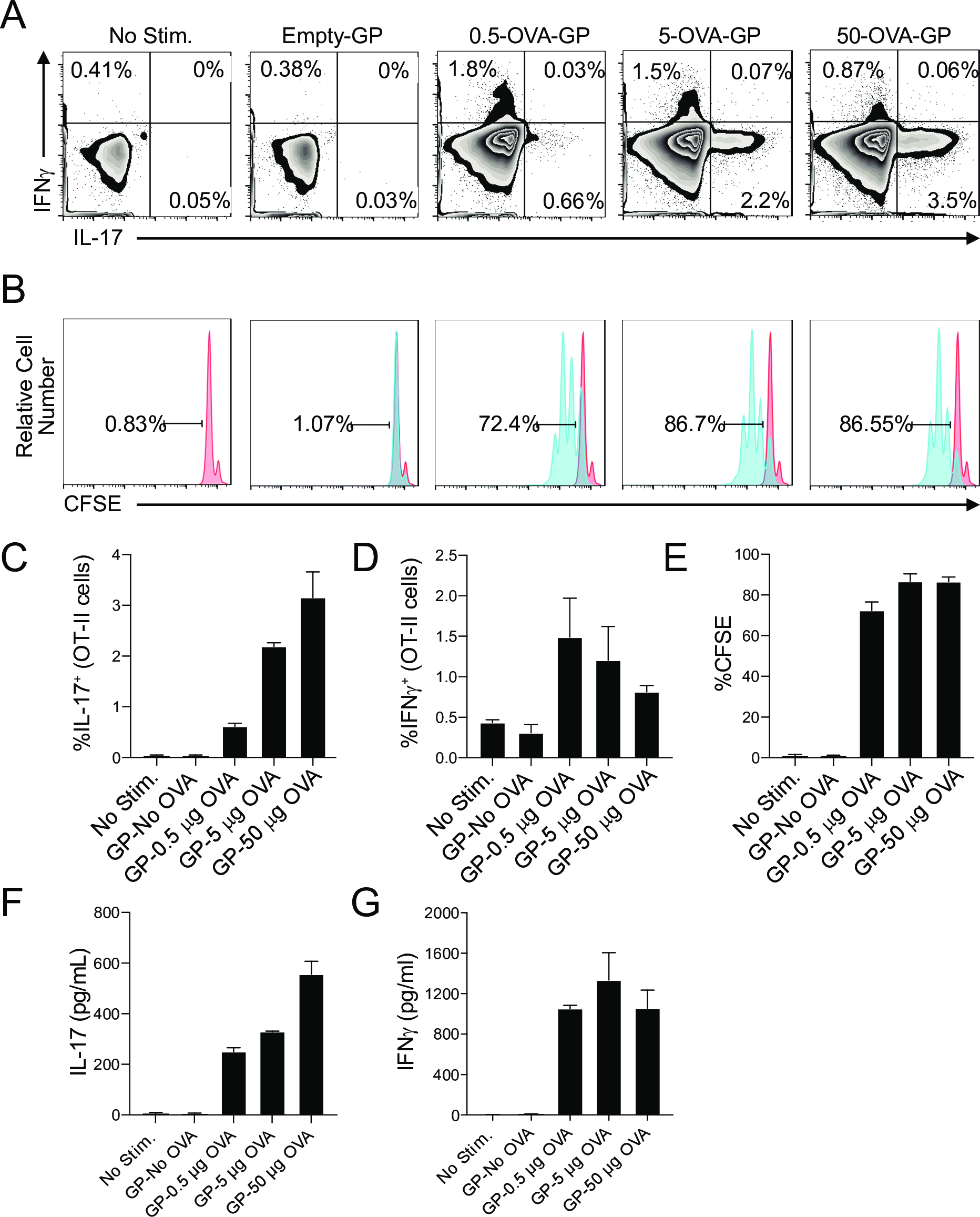

Supplement: S2 Fig — (A-G) BMDCs stimulated with GP-No OVA or GP-OVA (loaded with various concentrations of OVA) were used to activate naïve CFSE-labeled OT-II CD4+ T cells under non-polarizing conditions. (A) Representative flow cytometry plots show the degree of Th1 (IFNγ+) and Th17 (IL-17+) T cell differentiation under each condition of BMDC activation. (B) Representative flow cytometry histograms demonstrate the degree of T cell polarization corresponding to the data above with the pink histogram from the co-culture with no stimulation used as a reference point for the percentage of proliferation. (C-D) Bar graphs quantifying the percentage of IL-17+ (C) and IFNγ+ (D) OT-II cells and the percentage of CFSE proliferation (E). (F-G) Supernatants from the co-culture were collected on day 5 and analyzed for production of IL-17 (F) and IFNγ (G) by ELISA. Flow cytometry percentages and ELISA data and their standard deviation are representative of two replicates/condition. Data are representative of two experiments. (TIF) [file ppat.1008733.s002.tif]

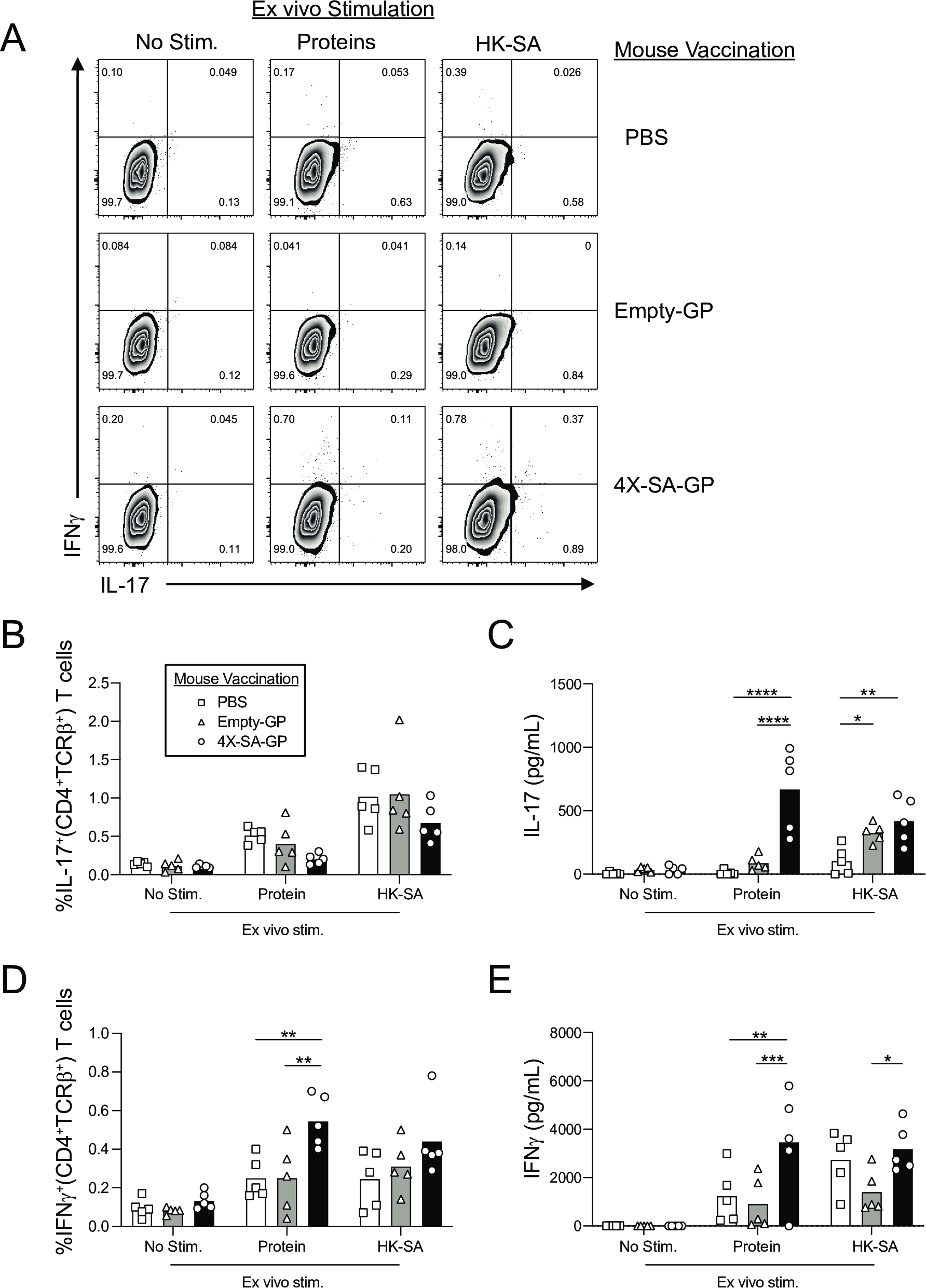

Supplement: S3 Fig — (A-E) Wild-type female mice were immunized one time with PBS (n = 5), Empty-GPs (n = 5), or 4X-SA-GP (n = 5). Five days after the vaccination, splenocytes from each mouse were stimulated with a mixture of all 4 S. aureus purified recombinant proteins (proteins) and heat-killed S. aureus (HK-SA) for 3 days. (No stim., no stimulation). After 3 days, the splenocytes were stimulated with phorbol myristate acetate (PMA) and ionomycin and then analyzed by flow cytometry after intracellular cytokine staining. Representative flow cytometry plots demonstrate the degree of IL-17 and IFNγ production from CD4+ T cells (gated on TCRβ+CD4+) in response to the stimuli (proteins, HK-SA) from the spleen (A). (B and D) Bar graphs show the frequency of IL-17 (B) and IFNγ positive cells in the spleen from the mice. Each data point represents an individual mouse in all of the bar graphs. (C and E) Supernatants were harvested at day 3 from the splenocyte (A) stimulation and the cytokines IL-17 (C) and IFNγ (E) were determined by ELISA. Data analysis was performed using ANOVA. *p<0.05, **p<0.01, ***p<0.005, ****p<0.0001. Data are representative of a single experiment. (TIF) [file ppat.1008733.s003.tif]

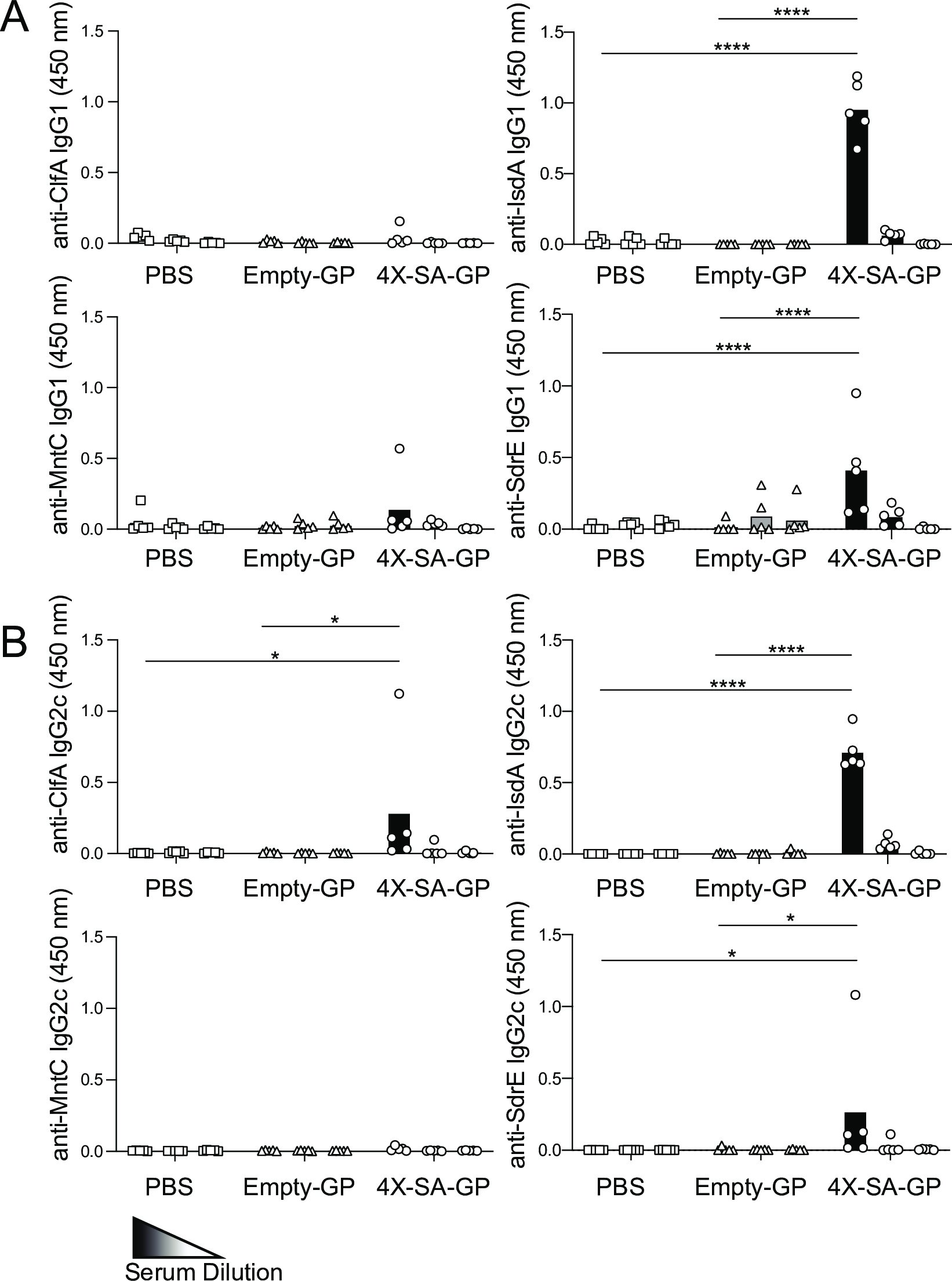

Supplement: S4 Fig — (A-B) Serum was collected from each group of vaccinated mice 2 weeks after one immunization with PBS, Empty-GP, or 4X-SA-GP (n = 5 mice per group). The serum was diluted 3 times at 1:1000, 1:10,000 and 1;100,000 and was then tested for antibodies specific for each of the 4 S. aureus proteins encapsulated in 4X-SA-GP by ELISA. The subclasses IgG1 (A) and IgG2c (B) were analyzed for specificity towards rClfA, rIsdA, rMntC, and SdrE. The read-out of the assay is the optical density (OD) at 450 nm for each serum sample. Each data point represents an individual mouse. Data analysis was performed using ANOVA. *p<0.05, **p<0.01, ***p<0.001, ****p<0.0001. Data are representative of a single experiment. (TIF) [file ppat.1008733.s004.tif]

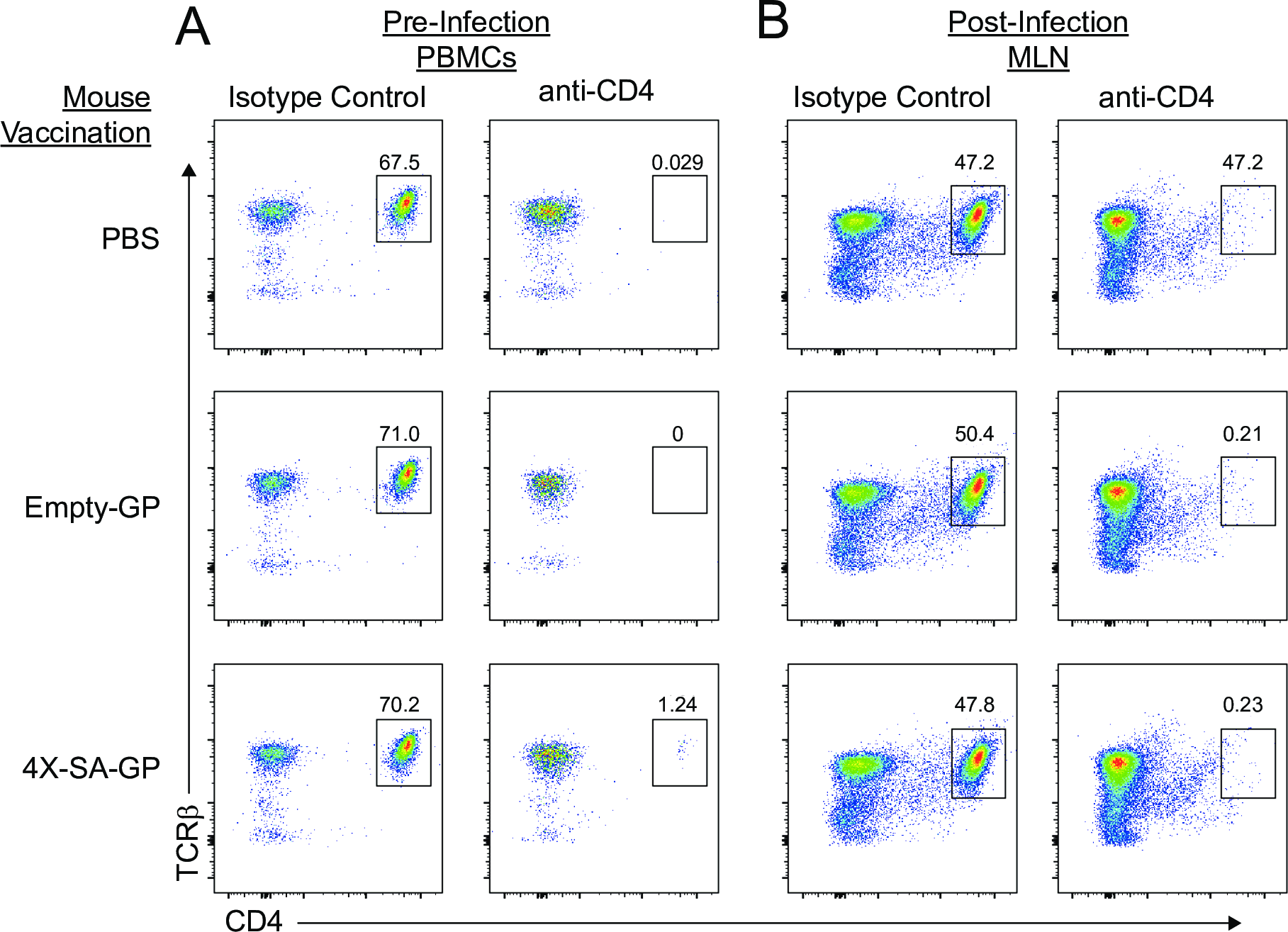

Supplement: S5 Fig — (A-B) Wild-type female mice were immunized once a week for 3 weeks with PBS (n = 9), Empty-GPs (n = 10), or 4X-SA-GP (n = 10). Four weeks after the final vaccination 4–5 mice per vaccination group were treated i.p. with anti-CD4+ antibody or the corresponding isotype control antibody on day -1 and day 0. On day 0, all groups of the mice were infected i.p. with 2x10^7^ CFUs of S. aureus (LAC USA300). (A) flow cytometry plots demonstrating the degree of CD4+ T cell depletion from pooled peripheral blood mononuclear cells (PBMCs) from each vaccination group of mice on day 0 before the mice were infected with S. aureus. (B) flow cytometry plots demonstrating the degree of CD4+ T cell depletion from pooled MLNs from each vaccination group of mice 24 hours after the mice were infected with S. aureus (day 1). Cells from (A) and (B) were gated on CD3+ cells. (TIF) [file ppat.1008733.s005.tif]

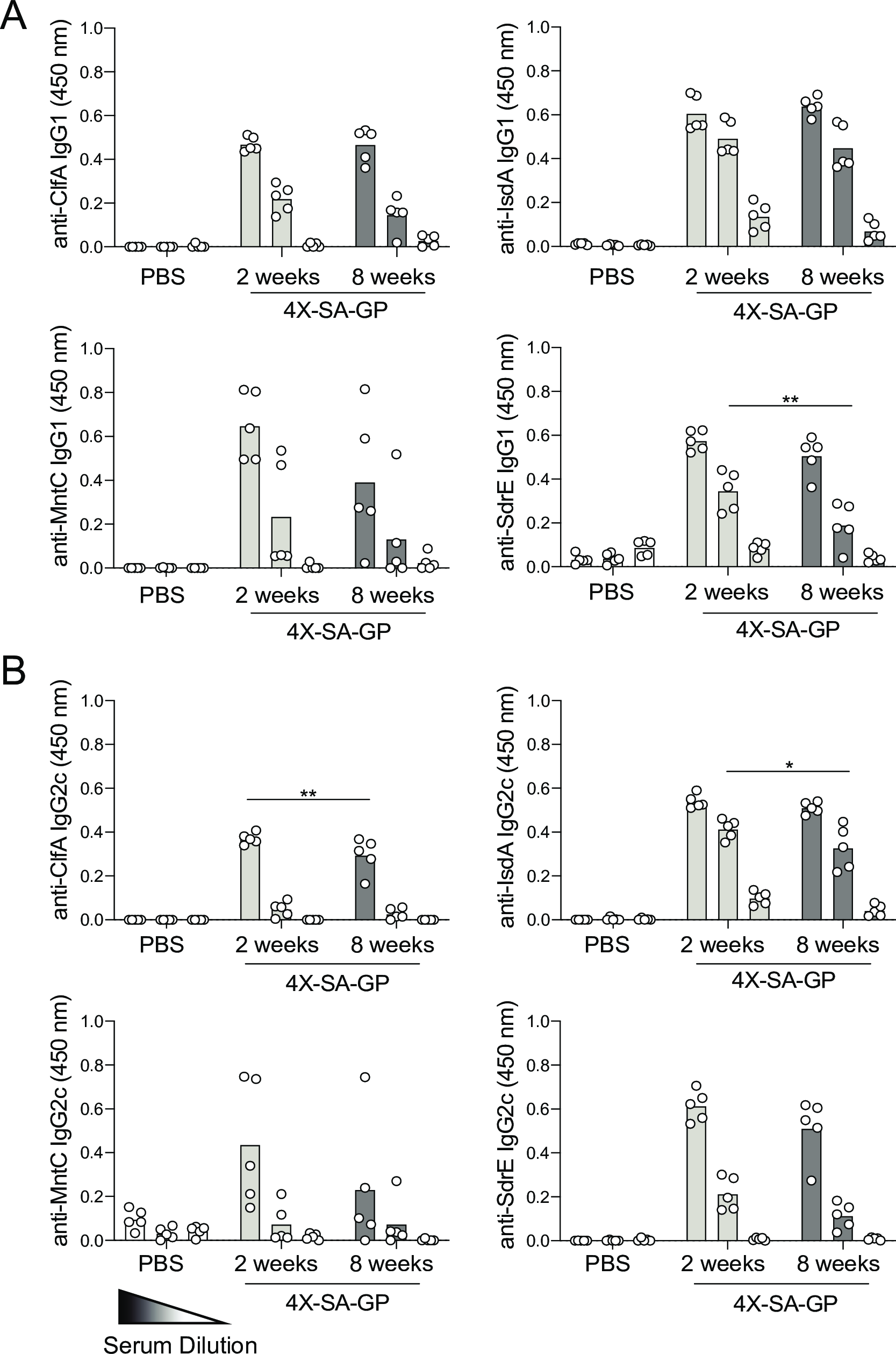

Supplement: S6 Fig — (A-B) Two sets of wild-type female mice were immunized once a week for 3 weeks with PBS (n = 5), Empty-GPs (n = 5), or 4X-SA-GP (n = 5). Serum was collected from one set of mice (PBS, Empty-GP, 4X-SA-GP; n = 5 mice/group) 2 weeks after the final vaccination and 8 weeks after the final immunization for the other set of mice (PBS, Empty-GP, 4X-SA-GP; n = 5 mice/group). The serum was diluted 3 times at 1:1000, 1:10,000 and 1;100,000 and was then tested for antibodies specific for each of the 4 S. aureus proteins encapsulated in 4X-SA-GP by ELISA. The subclasses IgG1(A) and IgG2c (B) were analyzed for specificity towards rClfA, rIsdA, rMntC, and rSdrE. The read-out of the assay is the optical density (OD) at 450 nm for each serum sample. Each data point represents an individual mouse. Data analysis was performed using ANOVA. *p<0.05, **p<0.005. Data are representative of at least two experiments for serum at two weeks and a single experiment for serum at 8 weeks. (TIF) [file ppat.1008733.s006.tif]
